# The antisymmetry of distortions

**DOI:** 10.1038/ncomms9818

**Published:** 2015-11-17

**Authors:** Brian K. VanLeeuwen, Venkatraman Gopalan

**Affiliations:** 1 Materials Research Institute and Department of Materials Science and Engineering, Pennsylvania State University, University Park, Pennsylvania 16802, USA

## Abstract

Distortions are ubiquitous in nature. Under perturbations such as stresses, fields or other changes, a physical system reconfigures by following a path from one state to another; this path, often a collection of atomic trajectories, describes a distortion. Here we introduce an antisymmetry operation called distortion reversal that reverses a distortion pathway. The symmetry of a distortion pathway is then uniquely defined by a distortion group; it has the same form as a magnetic group that involves time reversal. Given its isomorphism to magnetic groups, distortion groups could have a commensurate impact in the study of distortions, as the magnetic groups have had in the study of magnetic structures. Distortion symmetry has important implications for a range of phenomena such as structural and electronic phase transitions, diffusion, molecular conformational changes, vibrations, reaction pathways and interface dynamics.

## Abstract (web summary)

In a material system, a distortion constitutes a change from one stable state to another via a pathway built from a combination of atomic trajectories. Here, the authors present a distortion reversal antisymmetry operation which fully describes the symmetry of such pathways.

## Graphical abstract

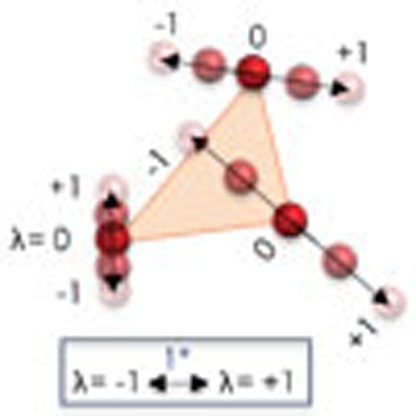

A distortion or distortion pathway refers to any one of the many possible paths between two or more states of a system. They are important for understanding chemical reaction kinetics^1^,^2^,^3^,^4^, phonon modes^5^,^6^,^7^, molecular pseudorotations and conformational changes^1^,^2^,^3^, diffusion^8^,^9^,^10^,^11^, the motion of interfaces such as grain boundaries^12^,^13^, domain walls^14^,^15^, and dislocations^16^,^17^, and emergent phenomena in transient or metastable states that arise from a distortion of the ground state^18^,^19^,^20^,^21^,^22^,^23^. There is often a privileged point on a pathway that is extremal in energy. For stable phonons, this is the ground state; for unstable phonons, the parent structure; for reaction pathways, the transition state; and for phase transitions, the saddle point. The relative energy of this point corresponds to the activation energy in the transition-state theory^1^,^24^. In many important pathways, it is found that the energies on either side of this privileged point are symmetric, for example, when opposite sides are mirror images of each other. An antisymmetry operation named distortion reversal, 1* is introduced here to describe the complete symmetry of such pathways. When combined with conventional symmetry groups, it gives rise to distortion groups. The symmetry of a distortion pathway is uniquely described by a distortion group.

Distortions, especially phonon modes, are studied today using representation analysis^5^,^6^,^7^, through decomposition onto a symmetry-adapted basis using irreducible representations (irreps). Why then is the concept of distortion-reversal symmetry and distortion groups necessary? It is instructive to look at the history, where a similar question has been posed for over 45 years regarding the need for time-reversal symmetry and magnetic groups versus representation analysis for the study of magnetic structures^25^,^26^. Opechowski and Dreyfus rigorously showed that representation analysis of magnetic structures and magnetic groups were equivalent, through a correspondence between one-dimensional real irreps and magnetic groups^25^,^27^,^28^. In practice, however, magnetic groups are widely used today due to their ease of use in describing and visualizing complex spin structures, easy transformations for predicting the form of magnetic property tensors and in deriving the energy invariants in magnetic crystals in a simple and transparent manner. In contrast, distortions and vibrations in molecules and crystals are studied today only by representation analysis. There is currently no equivalent formalism to the time-reversal symmetry or the magnetic groups for studying distortions. This work provides that framework through the introduction of distortion-reversal symmetry and distortion groups.

In developing distortion symmetry, we discovered that a somewhat similar concept was introduced several decades ago in transition-state theory in the limited context of reversing reactants and products in simple molecular reactions^1^,^2^,^3^. We demonstrate here that the concept of distortion groups is much more general, and can be used for studying distortions interpreted in a very broad sense, including phonon modes, the migration of crystal defects such as vacancies and interstitials, dislocation motion, ferroelectric switching, the motion of interfaces such as domain walls and grain boundaries, and electronic processes. Further, we show that they can predict the form of tensors that describe any property change of a system as a function of a general distortion parameter. We demonstrate that this symmetry framework can be applied not only to nuclear positions, but to the electronic structure itself, including the Berry phase of a distortion. We show that any distortion whose symmetry group includes distortion-reversing elements will have a Berry phase of exactly zero. Distortion symmetry can be applied to improve computational methods such as the popular nudged elastic band (NEB) method^29^, for finding minimum-energy pathways (MEPs). Applying distortion-reversing symmetries to NEB reduces the number of images required by a factor of two, which could increase performance significantly. Symmetry analysis can also identify numerical errors in NEB computations that may not be properly converged or symmetrized. The irreps of a distortion group classify the ways in which the symmetry can be broken by perturbations of the distortion pathway, potentially leading to lower-energy pathways than would be achieved by only applying NEB or related methods, such as CI-NEB. This is similar to how the irreps of the symmetry group of a structure can be used to classify and explore the types of stable and unstable phonons (perturbations of the nuclear positions) of the structure that can lead to other structures with lower energies. Double-antisymmetry groups can describe the symmetry of distortions of magnetic molecules and crystals, where both distortion-reversal and time-reversal antisymmetries become relevant.

## Results

### Definition of distortion-reversal symmetry

**Figure 1:**
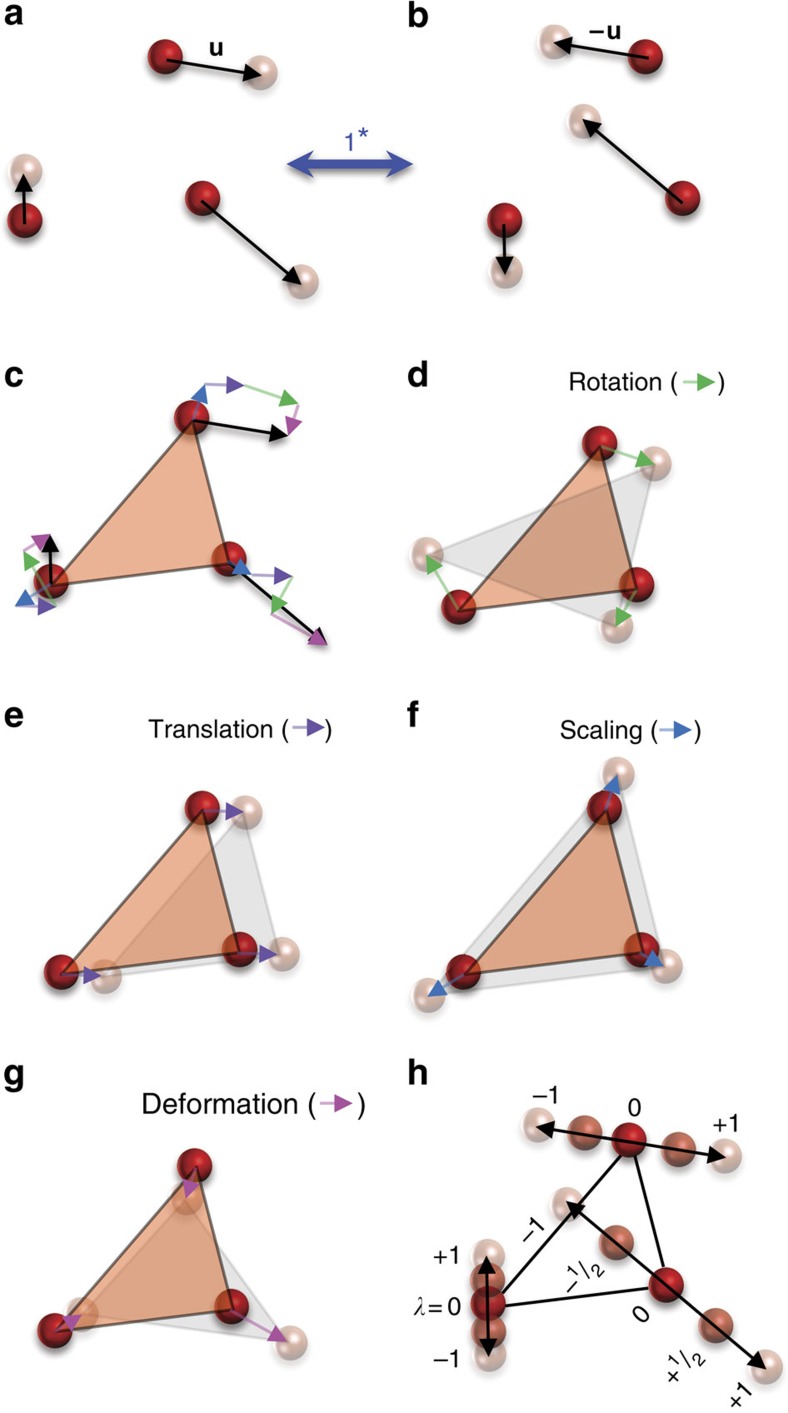
A simple example of a distortion and its decomposition. Three atoms (red spheres) are displaced by vectors **u** (black arrows) to their new positions (pink spheres) in **a**. The collection of the three displacement vectors, **u**, constitute a distortion. The distortion-reversal operation, 1*, reverses these displacements, **u**, and hence the distortion, as shown in **b**. The blue double-headed arrow indicates that repeated application of 1* can reverse between **a** and **b**. In **c**, each displacement vector, **u**, is decomposed into pure rotation (green arrows) (**d**), pure translation (purple arrows) (**e**), pure scaling (blue arrows) (**f**), and the remainder deformation (pink arrows) (**g**). As a guide to the eye, the orange and grey triangles in **c**–**g** indicate the initial and final configurations of the atoms, respectively. Linear atomic trajectories depicted here can be parameterized by −1≤*λ*≤1, as shown in **h**. Distortion-reversal operation, 1*, can thus be alternately viewed as a reversal of *λ* for a fixed **u**. This definition of 1* reversing the sign of *λ*, and not the reversal of displacement vectors, will be used in the rest of this article.

We introduce the concept of distortion reversal in Fig. 1a in a discrete system through three randomly placed atoms (in red) that form the parent structure. The atoms then displace to their new positions as per the displacements shown as arrows. The final distorted structure (in light pink) is the result of displacing each position accordingly. The action of the distortion-reversal operation, 1*, on the distortion in Fig. 1a is the reversal of displacements **u**_*i*_ of the atoms *i* (=1, 2, 3) to −**u**_*i*_ in Fig. 1b. These displacements have been decomposed in Fig. 1c into rotation (**u**_*i*,R_, Fig. 1d), translation (**u**_*i*,T_, Fig. 1e), scaling (**u**_*i*,S_, Fig. 1f) and deformation (**u**_*i*,D_, Fig. 1g), that is, **u**_*i*_=**u**_*i*,R_,+**u**_*i*,T_+**u**_*i*,S_+**u**_*i*,D_. This is analogous to the Helmholtz decomposition of continuous vector fields into components (see Methods). This decomposition is not necessary for implementing 1*, but it is helpful to illustrate the relationship between 1* and the rotation-reversal operation, 1^Φ^, introduced by Gopalan and Litvin^30^. While 1^Φ^ reverses the rotation component, **u**_*i*,R_ (Fig. 1e) to −**u**_*i*,R_, it has no clear implications for the other components. This creates a problem in implementing 1^Φ^, because it requires the identification of appropriate polyhedra within a structure that exhibits rigid rotations, but not the other components; the process for such polyhedral identification is non-unique, and often approximate in real systems. In this work, no such polyhedron is required to be identified in implementing 1* as seen from Fig. 1a,b. Further, 1* reverses all the components of **u**_*i*_, that is, 1*(**u**_*i*,R_, **u**_*i*,T_, **u**_*i*,S_, **u**_*i*,D_)=(−**u**_*i*,R_, −**u**_*i*,T_, −**u**_*i*,S_, −**u**_*i*,D_), not just rotation, **u**_*i*,R_, and in this sense, 1^Φ^ is a special case of 1*. There is an alternate way to view the action of 1* as described below, which will be used in the rest of this article. For linear atomic paths of atoms indexed by subscript *i*, the final atomic positions 
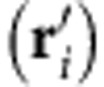
 are given by 
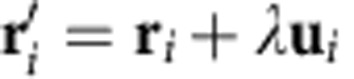
, where −1≤*λ*≤+1 and **r**_*i*_ are the initial positions of atoms, *i*, in the intermediate state (Fig. 1h). A typical distortion pathway may begin at *λ*=−1, go through an intermediate state at *λ*=0, and end at *λ*=+1. We can reverse this pathway by reversing the parameter *λ*→−*λ* while leaving the displacement amplitude **u**_*i*_ constant. The atomic trajectories in this example are linear with respect to *λ*, but in general, the pathway can be a nonlinear function 
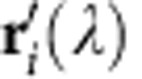
; the action of 1* will then be to reverse this function 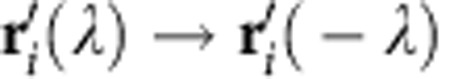.

We now make several important observations regarding the distortion-reversal operation, 1*. First, in addition to the ordinary dimensions, a distortion has a time-like dimension, *λ*, that describes the extent of the distortion. For a reaction pathway, this is the reaction coordinate; for a phonon mode, this is the amplitude; and for a phase transition, this is the order parameter. In this article, we normalize these over the range −1≤*λ*≤+1. Note that for coordinates (**r**, *t*, *λ*), spatial inversion, 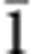, reverses the position vector **r**→−**r**, 1′ reverses the time *t*→−*t* and 1* reverses *λ*→−*λ*. Second, an analogy to the time-reversal operation, 1′ can be drawn from Fig. 1. If *λ* is replaced with *t*, then the displacement vectors **u** are replaced with velocity vectors, **v**, and 1* is replaced by 1′ between panels in Fig. 1a,b. If the velocities were decomposed in a similar way as in Fig. 1, the rotational component of this decomposition would correspond to the angular momentum, and for charged particles, magnetic moment. Because it was inspired by the practice of applying 1′ to reverse the localized magnetic moments of atoms, Gopalan and Litvin's rotation-reversal operation, 1^Φ^, focused exclusively on the rotational component^30^. Third, we note that the action of 1* is well defined on any structure that is parameterized by *λ*, not just a system of discrete atomic positions and displacements. For example, in calculating ferroelectric polarization, the modern theory of polarization implicitly parameterizes the electronic structure of a system by *λ* by parameterizing the ionic positions, and then calculating the ground-state electronic structure for a series of steps between 0≤*λ*≤+1 (ref. 31). On such a system, 1* has a well-defined action, even on the electronic structure itself (see later discussion on Berry phase). Fourth, we note that the symmetry of a distortion is not the symmetry of any particular static structure along the pathway, but rather of the symmetry of the entire pathway. The distortion group maps the entire pathway onto itself, and not the individual structures onto themselves; an infinitesimal section of the pathway may map to another section of the pathway through a distortion-reversing operation such that the pathway as a whole remains invariant.

### Application to molecular distortions

**Figure 2:**
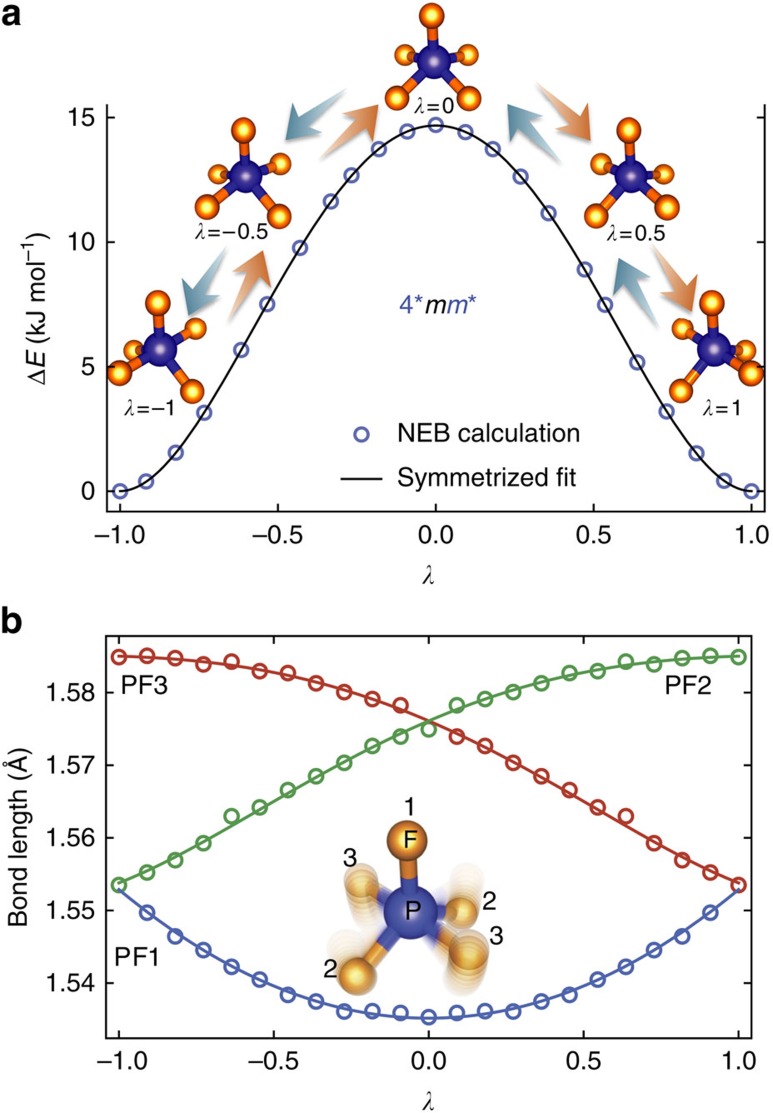
Distortion symmetry of the PF_5_ pseudorotation. The PF_5_ molecule undergoes pseudorotation from the *λ*=−1 state (left inset in **a**, blue atom is P and yellow atoms are F), through a transition state at *λ*=0 (middle inset in **a**), to the *λ*=+1 state (right inset in **a**). Pairs of static images at *λ* and −*λ* are related by a fourfold rotation along the PF1 bond (see inset in **b** for atom labels). The orange (light blue) arrows represent portions of the pathway going in the direction of increasing (decreasing) *λ*. The path segment over an infinitesimal path segment, *Δλ* to the left of *λ*=0 is transformed under 4 into the path segment −*Δλ* to the right of *λ*=0. The displacements of atoms between consecutive images on the left can be related to the displacements of atoms between consecutive images on the right. Thus 4 transforms images between *λ* and −*λ* and also the atomic displacements between consecutive images in such a way that the overall distortion path remains invariant. The set of all such operations that leave this pathway invariant form the complete distortion-symmetry group of this pathway, 4*mm*, where starred symmetries are distortion reversing and are highlighted by blue colour. The blue circles in **a** are energies from NEB calculations and the black line is the symmetrized fit as guaranteed by the 4*mm* symmetry group. The PF1 bond length (labelled in the inset in **b**, which shows the superimposed images of the molecule along the distortion path) as a function of *λ* for the NEB calculated path is plotted in **b** as blue circles; it is also guaranteed to be an even function with respect to *λ*, as is consistent with the symmetrized fit (blue line). Similarly, the PF2 (green circles and line) and PF3 (red circles and line) bond lengths are required by the 4*mm* symmetry to be mirror images of each other; this is consistent with the plot in **b**.

We first demonstrate distortion symmetry in a distortion of a simple molecule and show how it can predict relevant property changes. Figure 2 shows the pseudorotation distortion of phosphorus pentafluoride, PF_5_, a well-known fluxional molecule. The ground-state geometry of PF_5_ has 
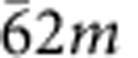
 symmetry. The distortion proceeds by the Berry mechanism^2^ where the pair of fluorine atoms on the high-symmetry axis move down as another pair of fluorine atoms move up. The structure goes through an intermediate transition state with 4 mm symmetry to a final state with 
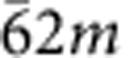
 symmetry. Although this distortion is not a rotation, the final state is equivalent to the original structure rotated by 90°, hence the term ‘pseudorotation'. We calculated the MEP using the NEB method^32^. The MEP represents the set of most likely trajectories that atoms will follow when physically transitioning between these states, and NEB calculations discretize the distortion pathway into a sequence of ‘images'. The highest-energy point on the MEP is known as the transition state and corresponds to *λ*=0 in Fig. 2. The energy of the transition state corresponds to the activation energy.

The MEP of the PF_5_ pseudorotation was determined to have 4*mm* symmetry (see the Methods section). A polynomial fit to the energy profile, Δ*E*, of the MEP, shown in Fig. 2a, is symmetric, that is, it is invariant under *λ*→−*λ*. This is required by the distortion group, 4*mm*, as shown next. Because 1* commutes with all spatial operations, the action of any starred symmetry operation on a coefficient of the power series expansion of *P*(*λ*) can be determined, where *P* is any property of the system. Specifically, the energy, *P*=Δ*E* in Fig. 2 is a scalar property and is invariant under rotation. By applying tensor transformation rules, we find that 4*Δ*E*(*λ*)=Δ*E*(−*λ*). However, since 4* is a symmetry operation, Neumann's principle^33^,^34^ states that 4*Δ*E*(*λ*)=Δ*E*(*λ*). Equating the two, one obtains


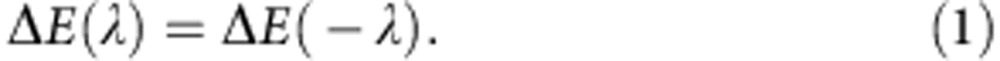


In other words, Δ*E*(*λ*) is a symmetric function of λ, which is consistent with the fit in Fig. 2. The three bond lengths, PF1, PF2 and PF3, also follow the requirements of the 4*mm* symmetry as shown in Fig. 2. For example, 4*(PF1(*λ*))=PF1(−*λ*), 4*(PF2(*λ*))=PF3(−*λ*) and 4*(PF3(*λ*))=PF2(−*λ*). Since 4* is a symmetry of the distortion, by Neumann's principle, PF1(*λ*)=PF1(−*λ*), PF2(*λ*))=PF3(−*λ*), and PF3(*λ*))=PF2(−*λ*). This is consistent with the results of the NEB calculation shown in Fig. 2b.

Supplementary Note 1 and Supplementary Fig. 1 present a similar application of distortion groups to molecular vibrations in H_2_O and NH_3_ molecules. For vibrations of NH_3_ corresponding to the doubly degenerate irrep, E, in particular, the distortion-symmetry framework is shown to be far more intuitive and transparent than the equivalent representation analysis. Distortion-reversal symmetry can be used to avoid the complexities of representation analysis such as in two- or higher-dimensional irreps. For many problems, distortion symmetry offers a simple and elegant alternative to traditional representation analysis.

### Application to finding an MEP

**Figure 3:**
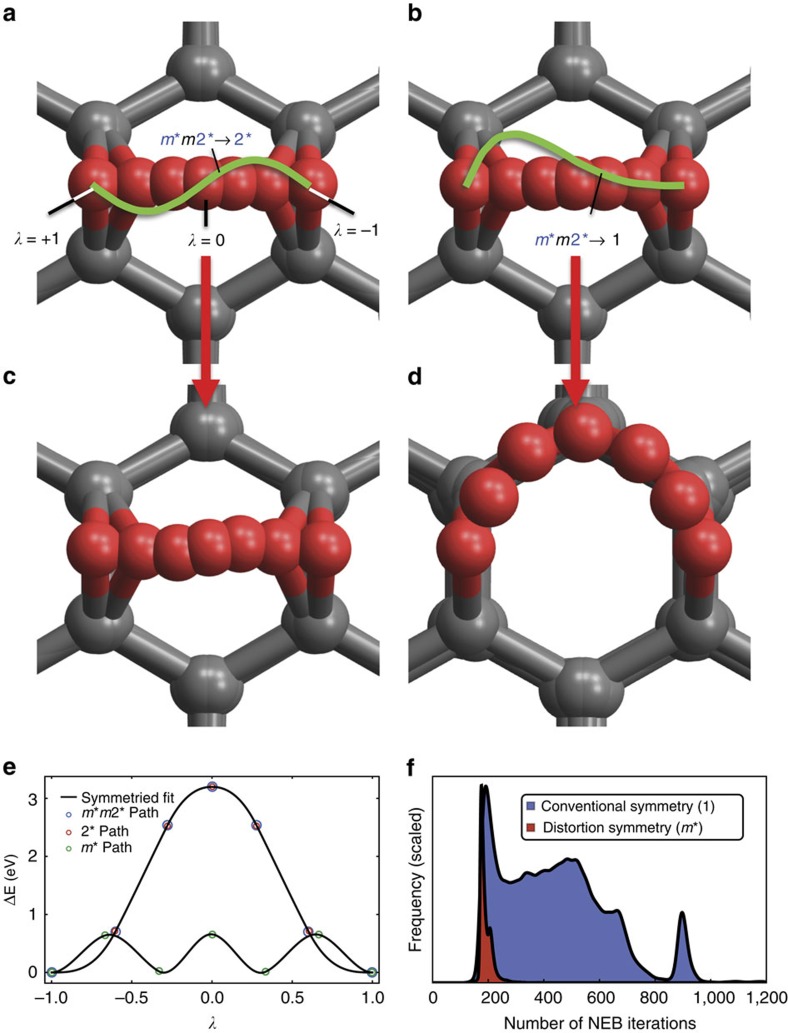
The consequences of distortion symmetry and balanced forces for NEB calculations. (**a**–**d**) Superimposed images along oxygen (red atom) diffusion paths on graphene (grey carbon atoms connected by grey bonds). In **a** and **b**, an initial linear path is assumed for the diffusion of a single oxygen atom from right (*λ*=−1) to left (*λ*=+1), across a C_6_ graphene ring. The symmetry of the path in **a** and **b** is *mm*2; the symmetry traps the path and prevents convergence to a minimum-energy pathway (MEP). To break the *mm*2 symmetry, we perturb this linear path as *mm*2→2 and *mm*2→1, respectively, as shown schematically exaggerated as green curves in **a** and **b** and indicated by the text in the inset. (**c**,**d**) The final paths after NEB relaxation starting from the perturbed paths of **a** and **b**, respectively, as indicated by red vertical arrows. The paths **c** and **d** have distortion symmetries of 2 and *m*, respectively. The 2 symmetry continues to trap the transition state (just like *mm*2 did for the linear path), whereas the initial path with trivial symmetry can correctly converge to a MEP with *m* symmetry. (**e**) The calculated energies of the images and the interpolation provided by QE's NEB module^45^. (**f**) Results for an example two-dimensional potential energy surface inspired by the above problem, using a simple NEB implementation. The plots are smoothed and rescaled histograms showing the frequency of NEB convergence at a given number of iterations in this example system for 100,000 randomly generated initial paths each with *m* or with trivial symmetry of 1. The two curves are rescaled to the same maximum height. Symmetrizing using the correct symmetry, *m* (red curve) reduced the number of NEB iterations needed in 98.97% of test cases. The average reduction was ∼2.3 × as compared with conventional symmetry (blue curve). This demonstrates that distortion symmetry can reduce the number of NEB iterations necessary for convergence.

Next, we demonstrate a symmetry-based approach to testing the stability of a pathway and checking the results of numerical computations for accuracy. This is demonstrated in the NEB calculation of activation energy for an oxygen atom diffusing across a C_6_ ring on the surface of graphene (Fig. 3a,b). Although not typically thought of as a ‘distortion', this diffusion path is a distortion within the symmetry framework presented in this article. Linear interpolation from the state with oxygen on the right (*λ*=−1), to the state with oxygen on the left (*λ*=+1) creates a path with m*m2* symmetry with a high-activation-energy barrier (Fig. 3a,b); this is not an MEP. Typically, only the first and last images are specified when setting up a NEB calculation and a linear path, such as this would be constructed by default by the NEB implementation. For example, this is the case for VTST Tools for VASP and neb.x for Quantum Espresso (QE). Relaxing this path using NEB cannot and does not change the m*m2* symmetry, because every NEB iteration must conserve distortion symmetry (Fig. 3c,d; Supplementary Note 2; Supplementary Figs 2–5), since the forces are balanced by symmetry. Without understanding that the symmetry needs to be broken, one might incorrectly conclude that the activation barrier for oxygen diffusion on graphene is several times larger than it actually is. We can now systematically explore perturbations to this path by using the irreps of m*m2* summarized by the character table given in Table 1.

For a distortion path discretized into *M* images with *N* atoms, the perturbations form a 3*NM*-dimensional space; this is similar to the 3*N*-dimensional space of phonons. Similar to the methods applied to studying phonons and in mode crystallography, we can use the irreps in Table 1 to construct a symmetry-adapted basis (Supplementary Note 1) from an arbitrary basis set for general perturbations of the path. Using perturbations associated with the irreps, Γ_2_, Γ_3_ and Γ_4_, we can reduce the symmetry of our initial guess path to the symmetry of their kernels, 2*, *m* and m* respectively. To achieve a trivial symmetry (point group 1) path, we can combine these. For the example in Fig. 3, subspaces associated with Γ_2_ and Γ_3_ are stable (Fig. 3c), while for the subspace associated with Γ_4_ (Fig. 3d), one or more directions are unstable, that is, small perturbations of the path in these directions will decrease the energy of the path and there will be a net force driving the path away from m*m2* and towards the m* path symmetry as seen in Fig. 3d. This is similar to the unstable phonons of an unstable structure. The stability or instability of any path perturbation could clearly be calculated using a method analogous to finite displacement methods used for calculating phonon frequencies of static structures. Using a symmetry-adapted basis for the perturbations of a distortion path would convey the same benefits as it does for phonon calculations, for example, reducing the force constants matrix to block diagonal form.

Figure 3e shows that the path with trivial symmetry (that is, point group 1) relaxes to a much lower-energy path with m* symmetry. The perturbations are exaggerated in Fig. 3; the maximum displacement of oxygen along the path was 0.1 Å in panel **a** and about 0.18 Å in panel **b**. Because NEB can only raise the symmetry of the path, not lower it (see Methods), the 2* path cannot achieve the same results and has approximately the same energy as the original relaxed m*m2* path. Essentially, the same path as our m*m2* path was studied by Dai *et al.*^35^ who reported a high-energy transition state with a barrier of 1.75 eV. Dai *et al.* also report a lower-energy transition state, apparently similar to our 0.66 eV state, but with 0.81 eV and an energy profile that is highly asymmetric with respect to the distortion coordinate. It thus erroneously violates the m* symmetry that our symmetry analysis in Fig. 3 indicates it must possess. Such unintentional errors are in fact quite common in literature as the survey examples in Supplementary Table 1 indicate. Supplementary Table 1 gives 50 examples of published studies where distortion symmetry would have been useful; this is clearly a very small subset of such studies. Distortion symmetry analysis can help identify errors and properly symmetrize computational results. We implemented a two-dimensional potential energy surface similar to the oxygen diffusion on graphene problem (see Supplementary Note 3; Supplementary Fig. 6 and Supplementary Software 1) to test whether there are potential efficiency gains in an NEB code by implementing distortion-reversal symmetry. The results shown in the histograms in Fig. 3f suggests that NEB codes can potentially converge in about half as many iterations if distortion symmetry is implemented. This benefit is in addition to the benefit of reducing the number of asymmetric images that would be necessary. Together, these two benefits may potentially speed up NEB convergence by a factor of four, that is, a fourfold reduction in total central processing unit (CPU) time, for paths with starred symmetry. Finally, our brief survey in Supplementary Table 1 suggests that a large number of references to the use of NEB in materials science are made in the study of pathways with energetically equivalent end points, such as in diffusion, dislocation, interface, and grain boundary motion, and ferroelectric and magnetic switching of domains^36^, and therefore may have distortion-reversing symmetries.

### Application to crystals and tensor properties

**Figure 4:**
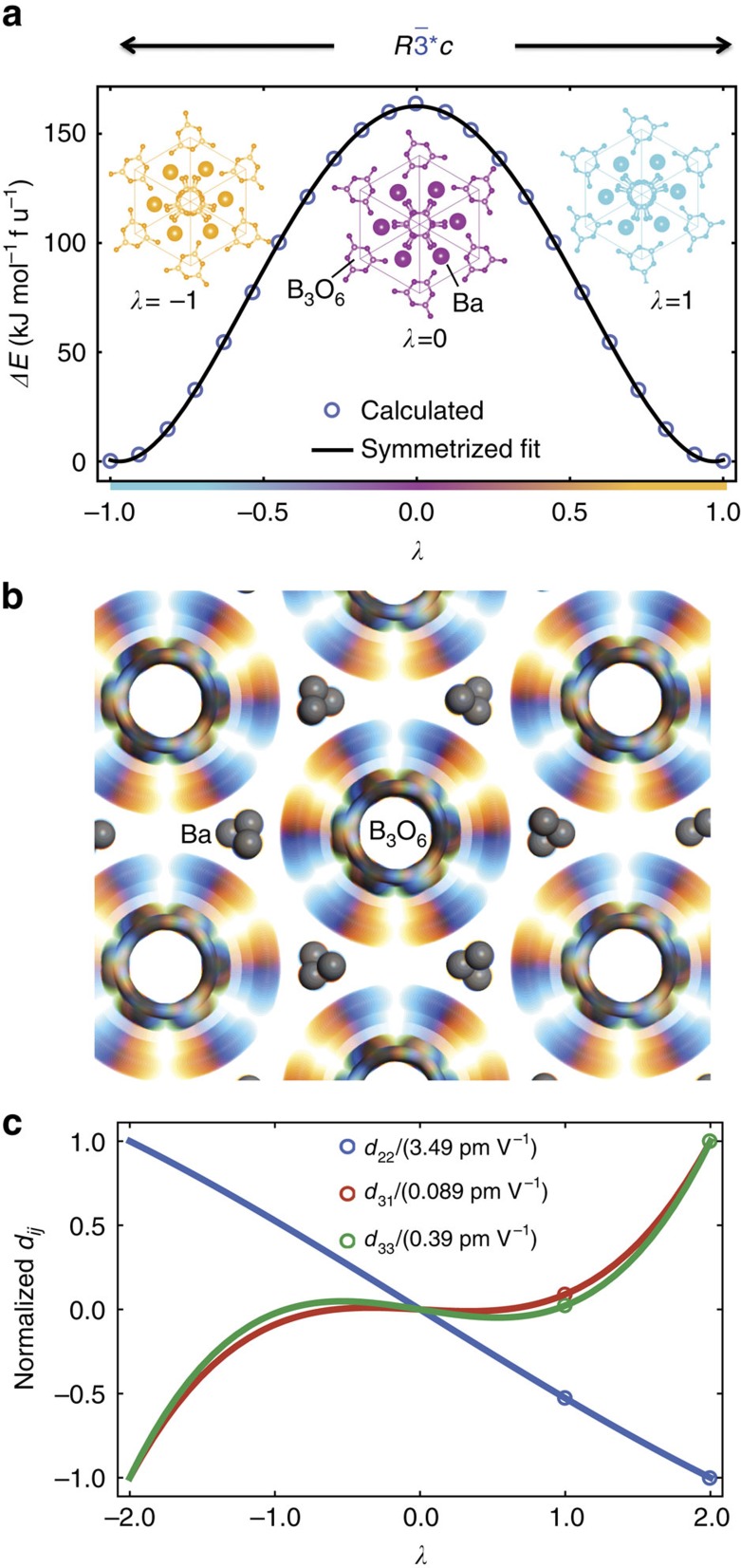
The application of distortion symmetry to a distortion of *β*-BaB_2_O_4_. The mostly rigid rotation of the B_3_O_6_ rings leads to two variants of *β*-BaB_2_O_4_ with *R*3*c* symmetry group, the *λ*=−1 variant (inset in orange in **a** and the *λ*=+1 variant (inset in cyan in **a**), transforming through a transition state at *λ*=0 (inset in magenta in **a**) with a symmetry of 
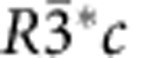
. The symmetry of this path, 
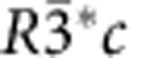
, requires that the energy profile in **a** is symmetric. (**b**) The superimposed images of *β*-BaB_2_O_4_ along the distortion pathway; their colour varies from orange, through magenta to cyan as *λ* varies from −1 through 0 to +1. (**c**) The optical second harmonic generation tensor coefficients along this pathway calculated by Cammarata and Rondinelli^37^ (red, green and blue circles) and a polynomial fit (red, green and blue lines) using only the coefficients that are consistent with 
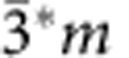
 point-group symmetry. Distortion symmetry predicts that these coefficients will be odd functions of the distortion parameter, *λ*, and zero when *λ=*0.

Next we demonstrate the application of distortion groups in predicting allowed energy couplings that are odd powers in the distortion parameter and may appear at first to be disallowed by conventional symmetry groups. We will use beta barium borate, *β*-BaB_2_O_4_, a widely used nonlinear optical crystal, as an example. Using a parent structure (*λ*=0) with 
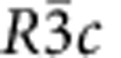
 symmetry^37^, we construct a distortion with *R*3*c* variants at *λ*=−1 and *λ*=+1. This distortion pathway has 
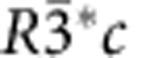
 symmetry. The calculated energy profile, Δ*E*(*λ*), is shown in Fig. 4a and is symmetric with respect to *λ*. This is a consequence of the starred symmetry operations, just as with the PF_5_ example. In Fig. 4b, we depict the sequence of intermediate structures along the distortion pathway by superimposing them using a colour scale. From the blurred pattern, we can see that this distortion is mostly the nearly rigid rotation of the B_3_O_6_ rings. For *β*-BaB_2_O_4_ and distortion group 
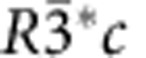
 (no. 4306 in the complete double-antisymmetry space group (DASG) listing^38^,^39^), the B_3_O_6_ rings are on the 12c site. From referring to the listing, this means that there are rings located at {0, 0, *z*}, {0, 0, −*z*+½}, {0, 0, −*z*} and {0, 0, *z*+½} with rotation vectors of [0, 0, *ω*_z_], [0, 0, *ω*_z_], [0, 0, −*ω*_z_] and [0, 0, −*ω*_z_], respectively. This tells us that the 
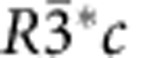
 symmetry requires that the rotation (*ω*) of the rings is only along the *z* axis and alternates every two rings along the column, that is, clockwise, clockwise, counterclockwise, counterclockwise and so on. The distortion symmetry listing also tells us that the displacement of the rings is only along the *z* axis and all the rings displace in the same direction with the same magnitude. This is just one of the many ways in which the concept of distortion symmetry can be used to make useful predictions about distortions.

It is certainly not intuitive *a priori* how properties, such as optical second harmonic generation (SHG), relevant to this material would vary with this distortion. The SHG interaction, 
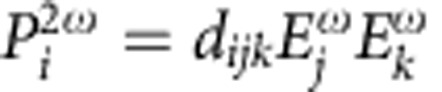
, creates a nonlinear polarization *P* at a frequency of 2*ω* by combining two photons with electric fields *E* at frequency *ω*. In Fig. 4c, we plot the calculated values (circles) for optical SHG coefficients for this crystal along the distortion pathway as calculated by Cammarata and Rondinelli^37^. The macroscopic point group of the *β*-BaB_2_O_4_ distortion described above is 
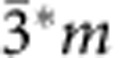
. We write *d*_*ijk*_ as a function of *λ* as,


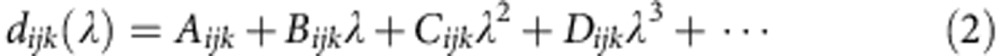



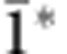
, an element of 
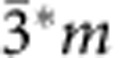
, combined with Neumann's principle, requires that *d*_*ijk*_(*λ*)=−*d*_*ijk*_(−*λ*). Thus, we immediately deduce that the function should be odd with respect to *λ*, and hence the even coefficients (*A*, *C* and so on) should be exactly zero. It also clearly implies that *d*_*ijk*_(0)=0. The points marked by open circles at *λ*=1.0 and *λ*=2.0 are from previously reported calculations^37^. The curves are the result of solving for *d*_*ijk*_(*λ*)=B_*ijk*_*λ*+D_*ijk*_*λ*^3^ that goes through these points. Since it was not obvious *a priori* that *d*_*ijk*_ should be an odd function of this distortion, this example demonstrates how applying distortion symmetry predicts the form of the tensors that describe the change in any property as a function of a distortion. This also suggests that in the Landau phenomenology, there should be an energy coupling of the form


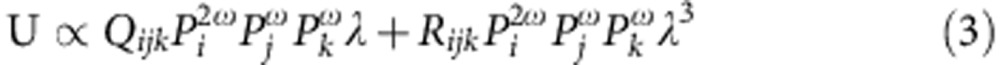


in the parent 
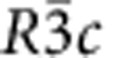
 (point group 
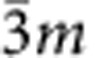
) phase. However, the polar third-rank tensors *Q*_*ijk*_ and *R*_*ijk*_ would be identically zero in the conventional 
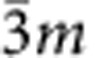
 parent phase as deduced by noticing that 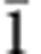 (*P*)=−*P* and 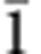 (*λ*)=*λ*. The only way such a coupling would exist is if the complete distortion symmetry of the pathway, namely, 
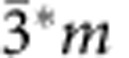
 is considered, since 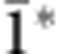
(*P*)=−*P* and 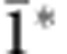
(*λ*)=−*λ*. This example shows the value of distortion symmetry analysis in easily revealing energy invariants that are odd powers in *λ*. It is far more transparent than the corresponding representation theory-based analysis.

Including distortion-reversing symmetry operations (that is, starred operations) can place greater restrictions on invariant property tensors. Table 2 compares the form of various types of tensors for a conventional symmetry group versus a group that includes starred operations corresponding to the distortion of the *β*-BaB_2_O_4_ given in Figure 4. Because of how the *A*_*ijk*_ and *C*_*ijk*_*λ*^2^ terms transform, *A*_*ijk*_ and *C*_*ijk*_ are 1*-even third-rank polar tensors. Likewise, *B*_*ijk*_ and *D*_*ijk*_ are 1*-odd third-rank polar tensors. From consulting Table 2, we find that the power series expansion of *d*_*ijk*_(*λ*) to the third power contains half as many degrees of freedom if the full symmetry group 
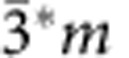
 is considered instead of only the unstarred symmetry of the distortion, 3*m*. If instead of the 
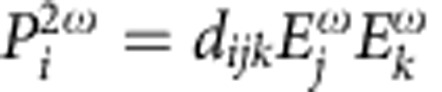
 interaction, we had considered 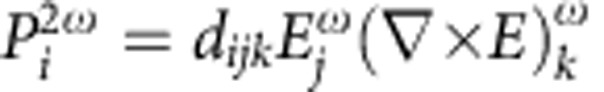 as an example, then the corresponding tensors *A*_*ijk*_ and *C*_*ijk*_ in an expansion similar to (2) would be axial 1*-even, 1′-even tensors, while the tensors *B*_*ijk*_ and *D*_*ijk*_ would be axial, 1*-odd, 1′-even tensors. Their forms and how they differ between the distortion group and conventional group is also given in Table 2. Thus, distortion symmetry can significantly reduce the number of tensor coefficients that are predicted to be non-zero.

### Application to diverse phenomena

**Figure 5:**
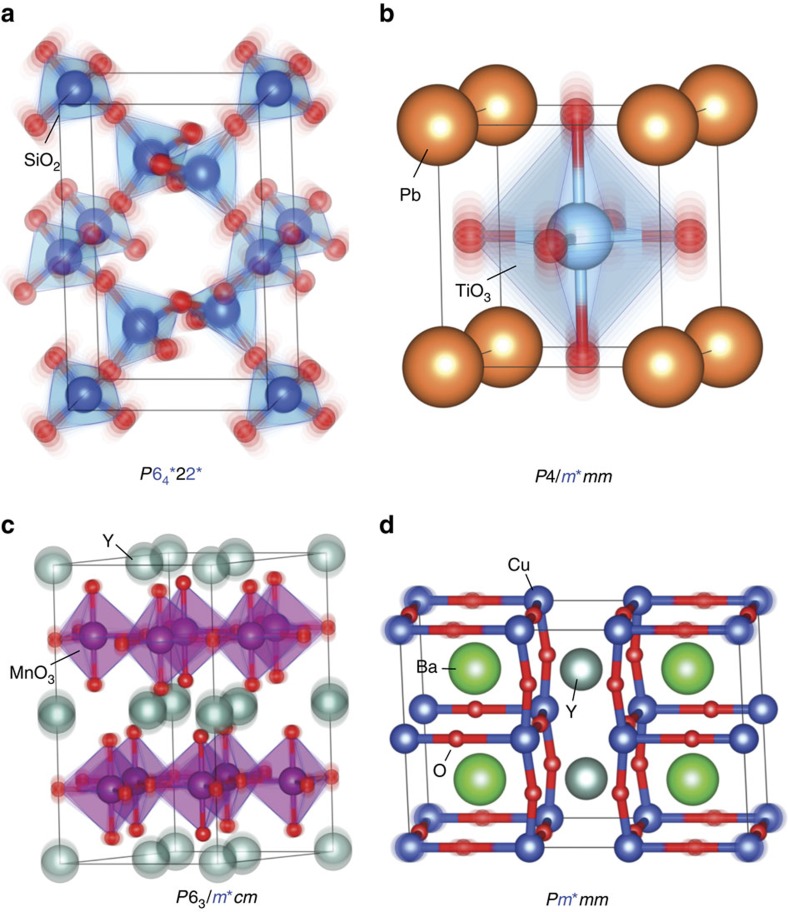
Four different example distortions in crystals and their distortion symmetry groups. Each panel depicts the superimposed structures through a distortion from *λ*=−1 to *λ*=+1 so that the movement of the atoms appears in the form of a blur. (**a**) A distortion pathway between two domain variants of a right-handed alpha quartz, passing through beta quartz at *λ*=0, with a distortion group of *P*6_4_22. (**b**) A distortion of ferroelectric PbTiO_3_ created by a linear interpolation between opposite (180°) polarization states; the pathway has a symmetry of *P*4/*mmm*. (**c**) A distortion of YMnO_3_ between opposite ferroelectric domain variants with a distortion symmetry of *P*6_3_/*mcm*. (**d**) A B_1u_ normal mode of YBa_2_Cu_3_O_6.5_ with a distortion symmetry of *Pmmm*.

The ubiquitousness of distortion symmetry is further illustrated in Fig. 5 with four examples. Each panel depicts the structures from *λ*=−1 to *λ*=+1 superimposed so that the movement of the atoms appears in the form of a blur. A common piezoelectric crystal quartz (SiO_2_) is depicted in Fig. 5a, where a distortion from one domain of right-handed alpha quartz (at *λ*=−1) through beta quartz (at *λ*=0) to another domain of right-handed alpha (at *λ*=+1) exhibits the distortion symmetry of *P*6_4_*22* (with point group 6*22*). Supplementary Note 4 and Supplementary Fig. 7 shows how there is an equivalent pathway in left-handed quartz with *P*6_2_*22* symmetry, as well as the symmetries of paths between left- and right-handed quartz. More generally, one can find distortion groups describing transformation between any two enantiomorphic structures (related by mirror) by choosing an appropriate parent that is intermediate between the two. Multiple such parents are possible, in principle. These ideas are also applicable to liquid crystals that can switch between left- and right-handed enantiomorphs under an electric field, a property that is utilized in display technologies.

A prototypical proper ferroelectric, PbTiO_3_ is depicted in Fig. 5b, where the distortion pathway runs between opposite polarization states and has *P*4/m*mm symmetry. An improper ferroelectric antiferromagnet, YMnO_3_, distorting from one ferroelectric domain, α^+^ at *λ*=−1 to the opposite domain α^−^ at *λ*=+1 exhibits a distortion symmetry of *P*6_3_/m*cm, as depicted in Fig. 5c. Note that the corresponding point groups (4/m*mm and 6/m*mm) for Fig. 5a,b allow for an energy invariant of the form *U*∝*P*.*λ*+*P*.*λ*^3^+.., where *P* is the polarization that develops under the distortion modes in question, parametrized by *λ*. In contrast, this coupling is zero under the conventional parent phase symmetries of *m*
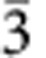
*m* and 6/*mmm*, respectively, again demonstrating the value of the distortion-reversal symmetry in revealing such couplings in a transparent and simple manner. This coupling in YMnO_3_ was confirmed by first-principles calculations^40^. Including antiferromagnetism and weak canted ferromagnetism in YMnO_3_ (ref. 41), we can consider two cases: either spins reverse or spins are invariant through α^+^→α^−^. The former has *P*6_3_′/m* symmetry and the latter has *P*6_3_′/*m*′* symmetry. Note that these DASGs involve two independent antisymmetries, 1* and 1′. A complete listing of the 17,803 DASGs has recently been made available by VanLeeuwen *et al.*^38^,^39^. These kinds of distortion pathways should exist in most ferroelectrics and multiferroics. One can analyse what pathways a domain wall could take in moving by a unit cell inside a ferroelectric, a ferromagnet or a multiferroic, using methods similar to the oxygen diffusion problem discussed in Fig. 3.

The 670 cm^−1^ B_1u_ mode of a superconductor, YBa_2_Cu_3_O_6.5_, is shown in Fig. 5d^18^. This mode has a distortion symmetry of Pm*mm, and has recently been shown to couple with A_g_ modes to create a transient structure that was reported to exhibit room-temperature coherent interlayer transport on picosecond timescales, reminiscent of superconductivity^19^,^20^. Including the coupling between this B_1u_ mode and the A_g_ modes retains the same distortion symmetry. The form of the invariant tensors describing changes in any property in these example systems as a function of distortion can be deduced, similar to that shown in Table 2.

### Application to the electronic structure and Berry phase

Finally, we show that distortion symmetry can be applied to the electronic structure of a distortion and has implications for Berry phase calculations. Ceresoli and Tosatti^42^ give the Berry phase along a path as:


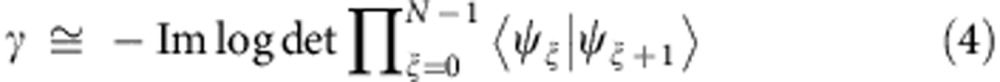


where *ξ*=0 through *ξ*=*N* are the indices of discrete images along the path (that is, each corresponds to a set of nuclear positions which can be used to compute the ground state electronic structure, *ψ*_*ξ*_). The product expressed in equation (4) for Berry phase expands as follows:


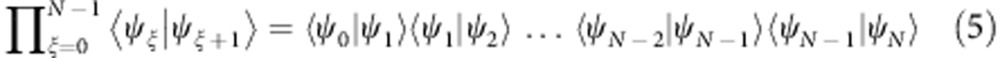


1* reverses the path such that the last image (at *ξ*=*N*) becomes the first image (at *ξ*=0) and vice versa. Thus:


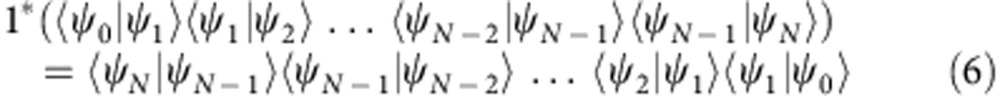


Substituting this into equation (4) for the Berry phase and simplifying leads to the conclusion that 1*γ=−*γ* for 
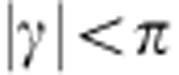
. Thus, if a distortion path is invariant with R* symmetry (that is, if R*γ=*γ*, for any Euclidean motion, *R*), then we conclude that


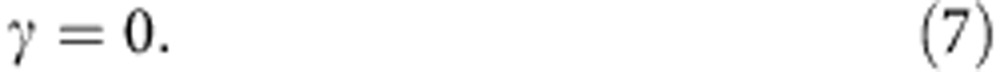


Thus, for a path with 1* symmetry, *γ*=0 (assuming 
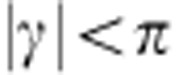
). Because *γ* is invariant under rotations, mirrors and translations (that is, Euclidean motions, *R*), this general result states that for any distortion pathway with a starred symmetry, R*, the Berry phase will be exactly zero. This includes all the example distortions given in this work that are described by distortion groups that contain any operation R*.

## Discussion

In the course of this study, we have come to conclude that distortion symmetry introduced here has applicability to a very wide range of physical problems, including atomic and electronic structure reconfigurations, reconfigurations of proteins and other biomolecules, motion of domain walls and grain boundaries, distortion tuning of metamaterials such as those exhibiting photonic bandgaps^43^, distortion-reversal symmetry protection of topological boundary modes^44^ analogous to time-reversal symmetry protection of topological insulators via Kramers theorem, the search for transient and metastable phases exhibiting emergent properties under a distortion^18^,^19^,^20^,^21^,^22^,^23^ and the search for intermediate stable structures in materials away from equilibrium, by reducing the asymmetric domain. The double-antisymmetry groups created from incorporating both distortion-reversal and time-reversal antisymmetries could be applied to explore the energy landscape of magnetic structures under a distortion. Similar to the impact of time-reversal antisymmetry and magnetic groups, we foresee a commensurate impact of distortion-reversal antisymmetry and distortion groups on a diverse set of problems and design tools used in the physical sciences.

## Methods

### The decomposition of a vector field

The decomposition applied to the simple distortion seen in Fig. 1 was performed by selecting a basis for translation, rotation, scaling and deformation components. Supplementary Fig. 1 shows an example of such a basis for a water molecule and, in this case, it is also symmetry adapted. After the basis is selected, the displacement vectors are projected onto it and each component can be isolated as shown in Fig. 1. This and other notions of decomposition into rotational and non-rotational components were explored in our attempts to formalize the concepts of rotation-reversal symmetry.

### PF_5_ pseudorotation NEB calculations

The PF_5_ pseudorotation MEP was computed using DMol^3^ in Materials Studio 6.0 (ref. 32). The approximate structure was input using Materials Studio's tools and then geometrically optimized using DMol^3^. This structure was taken as the *λ*=−1 variant and was copied and rotated 90° around the high-symmetry axis to make the *λ*=+1 variant. Then the Reaction Preview tool was used to match the atoms of the structures and generate an initial guess path. This guess was used as input for the DMol^3^ Transition State Search tool whose output was then run in the Transition State Conformation Tool, which performs NEB to find a MEP. The output from NEB was symmetrized to remove the small asymmetric numerical errors and used to construct the plots in Fig. 2.

### Oxygen diffusion on graphene NEB calculations

The geometrically optimized structure was from an example calculation used at the QE2014 workshop held at PennState. The *λ*=−1 structure consists of a 3 × 3 supercell of graphene with an oxygen atom bonded to the surface, as part of an epoxy functional group. This was mirrored to create the *λ*=+1 structure. These structures were used as the first and final images in the input for QE's NEB module (neb.x)^45^. Seven images were used. These are linearly interpolated from the first and final images. This initial guess path, discretized into a chain of seven images, relaxed into the path seen in Fig. 3a and Fig. 3b with m*m2* symmetry.

Next, two new paths were created from the m*m2* path using small symmetry-breaking perturbations of the oxygen trajectory parallel to the graphene sheet. The first was a sinusoidal perturbation with an amplitude of 0.1 Å resulting in a path with 2* symmetry. The second was a perturbation of −(*λ*^5^−5*λ*^4^−6*λ*^3^+2*λ*^2^+7*λ*+3)/32 Å resulting in a path with only trivial symmetry (that is, point group 1). These two new paths were then relaxed using QE's NEB module again to get the paths shown in Fig. 3c,d.

The reason that starred symmetry operations affect the results of NEB calculations in this way is because NEB commutes with 1* in the same way that conventional symmetry operations commute with physical laws. Clearly, NEB(X) gives the same result as A^−1^NEB(AX) where X is the initial guess path and A is an ordinary symmetry operation, such as a rotation or a mirror. Similarly, NEB(X) gives the same result as 1*^−1^NEB(1*X) and, since 1*^−1^=1*, NEB(X) gives the same result as 1*NEB(1*X). This is no different from the idea that physical laws should not depend on what basis one chooses for their coordinate system. If A* is a symmetry of the initial guess path, that is, X=A*X, then, by substitution, NEB(X)=A*NEB(X). Thus, due to the commutativity of A* with NEB, X=A*X implies NEB(X)=A*NEB(X), that is, a symmetry of the initial guess will also be a symmetry of the results. In practice, however, A^−1^NEB(AX) is not exactly equal to NEB(X), because the NEB implementation will have small symmetry-breaking numerical errors.

### *β*-BaB_2_O_4_ (BBO) calculations

The *β*-BaB_2_O_4_ distortion shown in Fig. 4 was created using Materials Studio's Reaction Preview tool by matching atoms of a *β*-BaB_2_O_4_ variant with its inverted variant. The result is a path that goes through an 
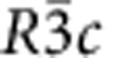
 intermediate, as shown in Fig. 4a,b. The energy along this path, as plotted in Fig. 4a, was computed using Materials Studio's CASTEP module and symmetrized to remove small asymmetric numerical errors. Similar methods were applied to make the energy plot for the quartz example in Supplementary Fig. 7.

Our *β*-BaB_2_O_4_ distortion path is similar, but not identical, to the linear path implied by Cammarata and Rondinelli^37^ where the displacements from the hypothetical 
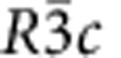
 parent structure are scaled by a factor. In particular, we note that our path has rigid or near-rigid rotation of the B_3_O_6_ rings, whereas linearly scaling the displacements creates a path that diverges from rigid rotation as rotation angle increases. Nonetheless, as the two paths are still very similar and have the same distortion symmetry, we have used the results of Cammarata and Rondinelli^37^ to create Fig. 4c.

### Method for determining the distortion symmetry group

Let *S*(*λ*) denote the structure at *λ*. Let *G*(*λ*) denote the conventional symmetry group of *S*(*λ*). If there exists *A*∈*G*(*λ*=0) such that *AS*(*λ*)=*S*(−*λ*) for all −1≤*λ*≤+1, then the symmetry of the distortion is 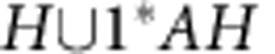, where 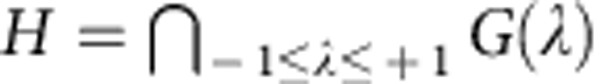. Otherwise, *H* is the symmetry of the distortion. In other words, find the conventional symmetry group of all the images in a pathway (*λ* from −1 to +1); the intersection of these groups is the group *H*. Now find an element *A* in the conventional symmetry group of the structure at *λ*=0 that can transform a structure at *λ* to a structure at −*λ*. The distortion group of the pathway is then 
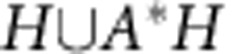
. If no such *A* can be found, then *H* is the symmetry of the distortion pathway.

## Additional information

**How to cite this article:** VanLeeuwen, B. K. & Gopalan, V. The antisymmetry of distortions. *Nat. Commun.* 6:8818 doi: 10.1038/ncomms9818 (2015).

## Supplementary Material

Supplementary Information Supplementary Figures 1-7, Supplementary Table 1, Supplementary Notes 1-4 and Supplementary References.

Supplementary Software 1 A Mathematica file referenced in Supplementary Note 3.

## Figures and Tables

**Table 1:** The character table of the distortion group *m* *m*2.

	**1**	2*	***m***	m*	**Kernel**
**Γ_1_**	1	1	1	1	m*m2*
**Γ_2_**	1	1	−1	−1	2*
**Γ_3_**	1	−1	1	−1	*m*
**Γ_4_**	1	−1	−1	1	m*

The four one-dimensional irreducible representations (irreps), Γ_1_–Γ_4_, represent four distinct types of perturbations to a distortion pathway with m*m2* symmetry. Each irrep has a kernel symmetry shown in the last column.

**Table 2:** Tensor forms for 
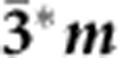
 versus 3*m* symmetry groups.

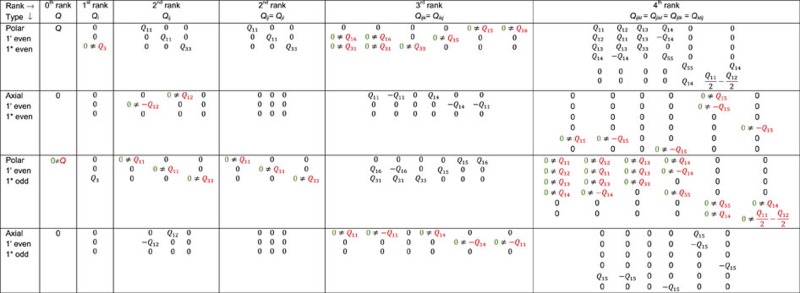

A comparison of the form of various general tensors of different ranks (columns) and types (rows) that are predicted by the distortion group 
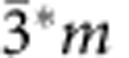
 versus that predicted by the conventional 3*m* symmetry group, which are the distortion symmetry and conventional symmetry groups, respectively, of the distortion path for *β*-BaB_2_O_4_. For example, the second row, sixth column corresponds to the form of a general third-rank polar tensor, *Q*_*ijk*_ (*i*,*j*,*k* correspond to an orthogonal crystal physics coordinate system) that is invariant under time reversal, 1′, and distortion reversal, 1*. The tensor coefficients shown in colour are the predictions that differ between the distortion symmetry (in green) versus the conventional symmetry group (in red). The remaining coefficients in black correspond to predictions that are identical between the two symmetry groups. Four other tensor types, namely, polar 1′ odd 1* odd, polar 1′ odd 1* even, axial 1′ odd 1* odd and axial 1′ odd 1* even, have identical tensor forms for the two symmetry groups, and hence are not shown.
