# Supplementary material for: The antisymmetry of distortions
Source: Nat Commun. 2015 Nov 17;6:8818. doi: 10.1038/ncomms9818 (PMC4660051; doi:10.1038/ncomms9818)
Supplement: Supplementary Information — Supplementary Figures 1-7, Supplementary Table 1, Supplementary Notes 1-4 and Supplementary References. [file ncomms9818-s1.pdf]

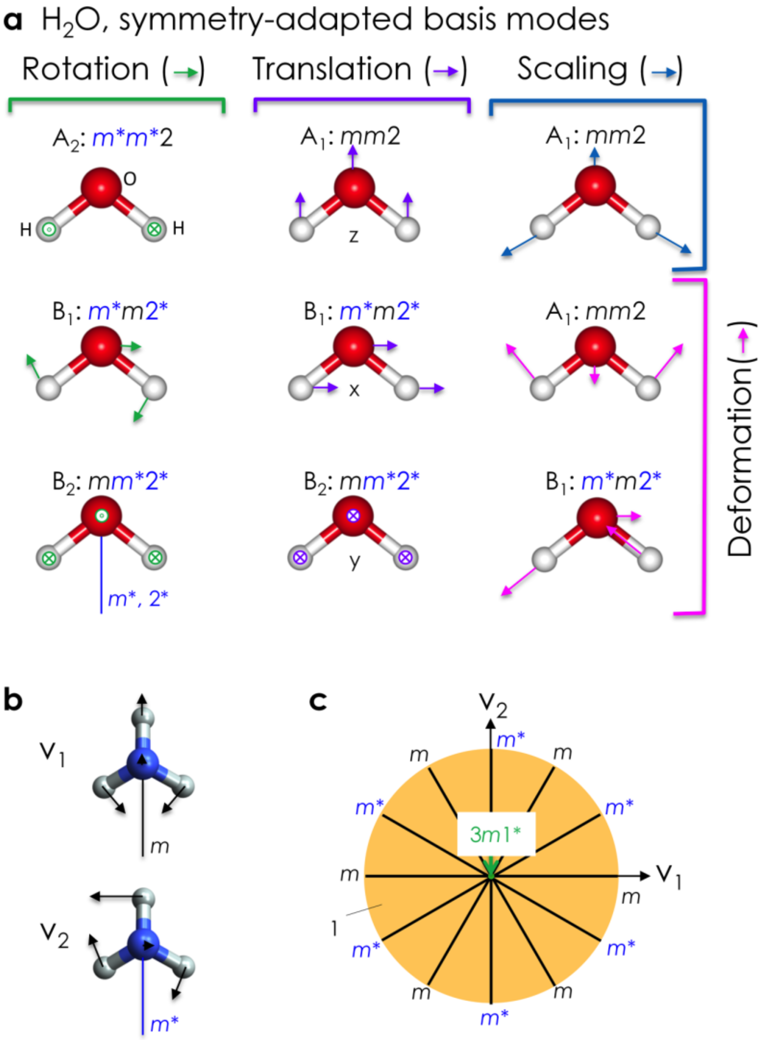

Supplementary Figure 1 | A comparison of representation analysis and distortion symmetry for simple molecules. Water, H_2O, has 3 atoms, each with three degrees of freedom of motion, hence 9 possible modes. A symmetry adapted basis is given in panel **a** for the atomic displacements of H_2O for these 9 modes, classified into pure rotations (green arrows), pure translations (purple arrows), pure scaling (blue arrows), and the remainder deformation (pink arrows). A doubly degenerate vibrational mode of ammonia molecule, NH_3, can be mapped to displacement vector basis, V_1 and V_2 , exhibiting distortion reversal symmetries of *m* and *m** respectively, as depicted in panel **b**. The distortion space formed by linear combinations of vectors V_1 and V_2 is depicted in panel **c**, and symmetry groups for various combinations is indicated.

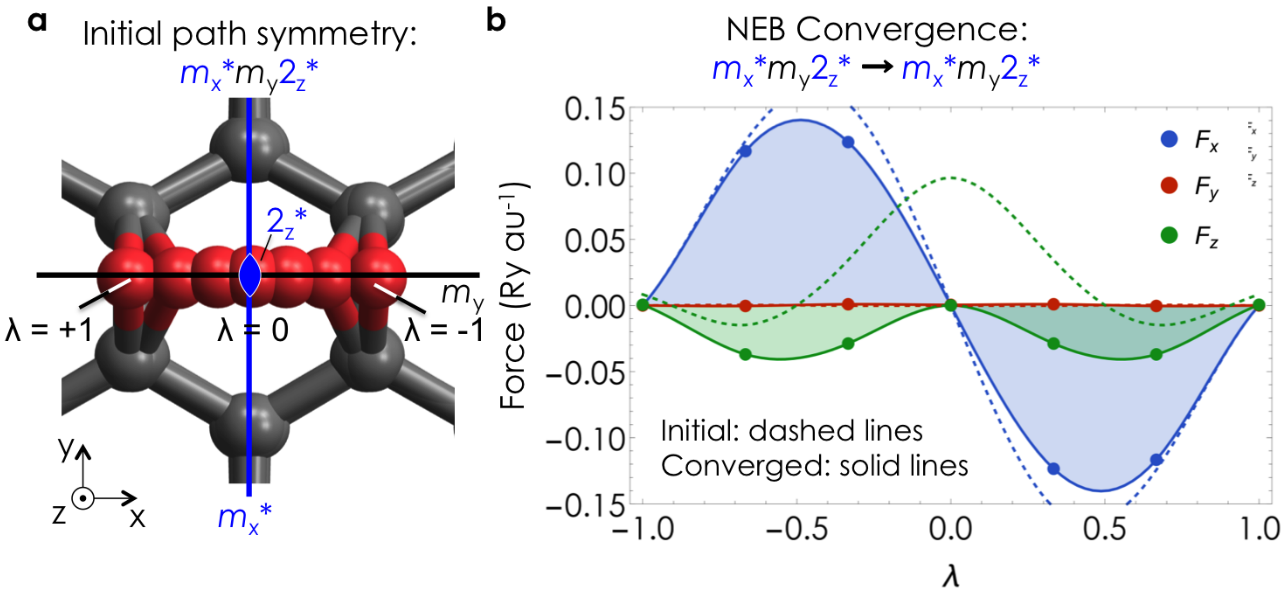

Supplementary Figure 2| Electrostatic forces on oxygen for the *m*m2 diffusion path across a graphene C_6 ring.** Panel **a** depicts superimposed images along an oxygen (red atom) diffusion path on graphene (grey carbon atoms connected by grey bonds). An initial linear path is assumed for the diffusion of a single oxygen atom from right (λ=-1) to left (λ=+1), across a C_6 graphene ring as shown by the trajectory of the superimposed red atoms. This linear path has m^*m2^* symmetry; the symmetry elements of this group are overlaid on the image. Panel **b** plots the x, y, and z components of the force on oxygen for the initial path (dashed lines) and the final NEB converged path (solid with axis filling). The forces of one NEB iteration are used to update the positions for the next iteration, thus guaranteeing that the $m_x^*m_y2_z^*$ symmetry cannot be broken in any subsequent iteration.

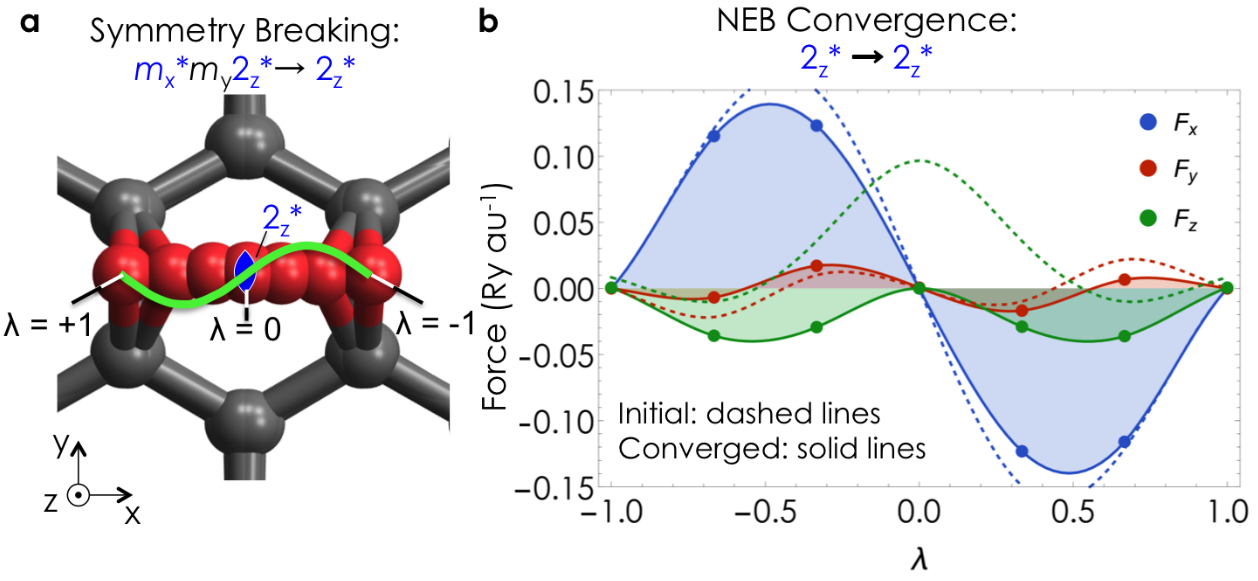

Supplementary Figure 3| Electrostatic forces on oxygen for the 2* perturbed diffusion path across a graphene C_6 ring. Panel **a** depicts superimposed images along an oxygen (red atom) diffusion path on graphene (grey carbon atoms connected by grey bonds). An initial linear path is assumed for the diffusion of a single oxygen atom from right (λ=-1) to left (λ=+1), across a C_6 graphene ring as shown by the trajectory of the superimposed red atoms. This linear path has *m***m*2* symmetry. The green curve indicates an exaggerated perturbation with 2_z* with its symmetry diagram overlaid. Panel **b** plots the *x*, *y*, and *z* components of the force on oxygen for the initial path (dashed lines) and the final converged path (solid with axis filling). The 2_z* symmetry is consistent with the forces on both the initial and converged paths. The 2_z* symmetry in the initial guess prevents NEB iterations from finding a significantly lower energy transition state (TS).

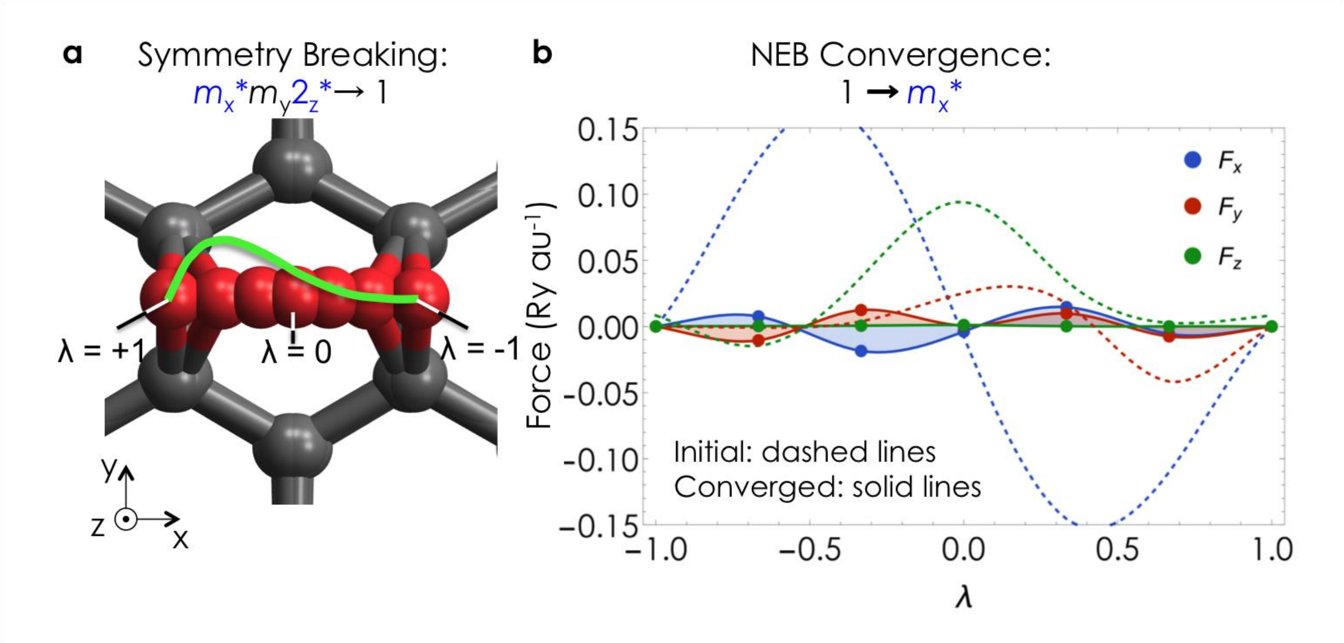

Supplementary Figure 4| Electrostatic forces on oxygen for the trivial symmetry perturbation for diffusion path across a graphene C_6 ring. Panel **a** depicts superimposed images along an oxygen (red atom) diffusion path on graphene (grey carbon atoms connected by grey bonds). An initial linear path is assumed for the diffusion of a single oxygen atom from right (λ=-1) to left (λ=+1), across a C_6 graphene ring as shown by the trajectory of the superimposed red atoms. This linear path has m^*m2^* symmetry. The green curve indicates an exaggerated perturbation with a trivial symmetry of 1. Panel **b** plots the *x*, *y*, and *z* components of the force on oxygen for the initial path (dashed lines) and the final converged path (solid with axis filling). NEB iterations drive the path to the much lower energy m^* path.

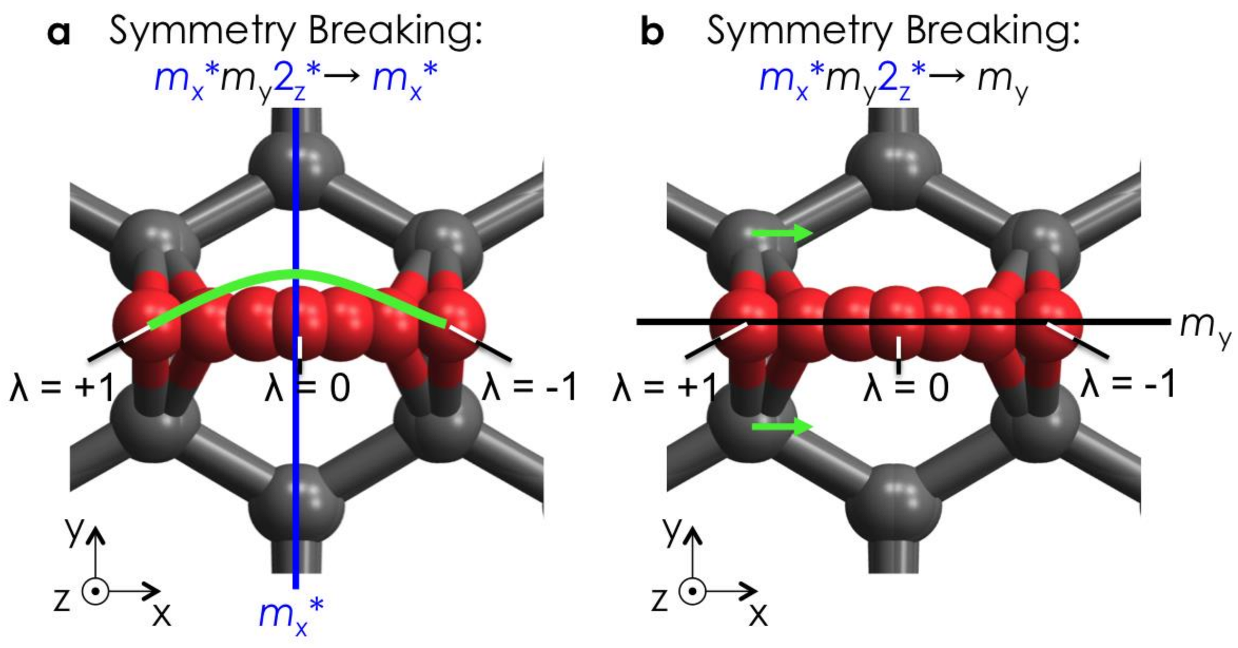

Supplementary Figure 5| Electrostatic forces on oxygen for the *m and *m* perturbations for diffusion path across a graphene C_6 ring.** Panel **a** and **b** depict superimposed images along oxygen (red atom) diffusion paths on graphene (grey carbon atoms connected by grey bonds). An initial linear path is assumed for the diffusion of a single oxygen atom from right (λ=-1) to left (λ=+1), across a C_6 graphene ring as shown by the trajectory of the superimposed red atoms. This linear path has a distortion symmetry of *m***m*2*. The green curves indicates an exaggerated perturbation with a symmetry of m_x* and m_y respectively. Panel **b** plots the *x*, *y*, and z components of the forces on oxygen for the initial path (dashed lines) and the final converged path (solid with axis filling). NEB iterations drive the path in **a** to the much lower energy *m** path. The m initial path does not converge to the low energy *m** path because *m* is not a subgroup of *m** and NEB iterations must conserve distortion symmetry.

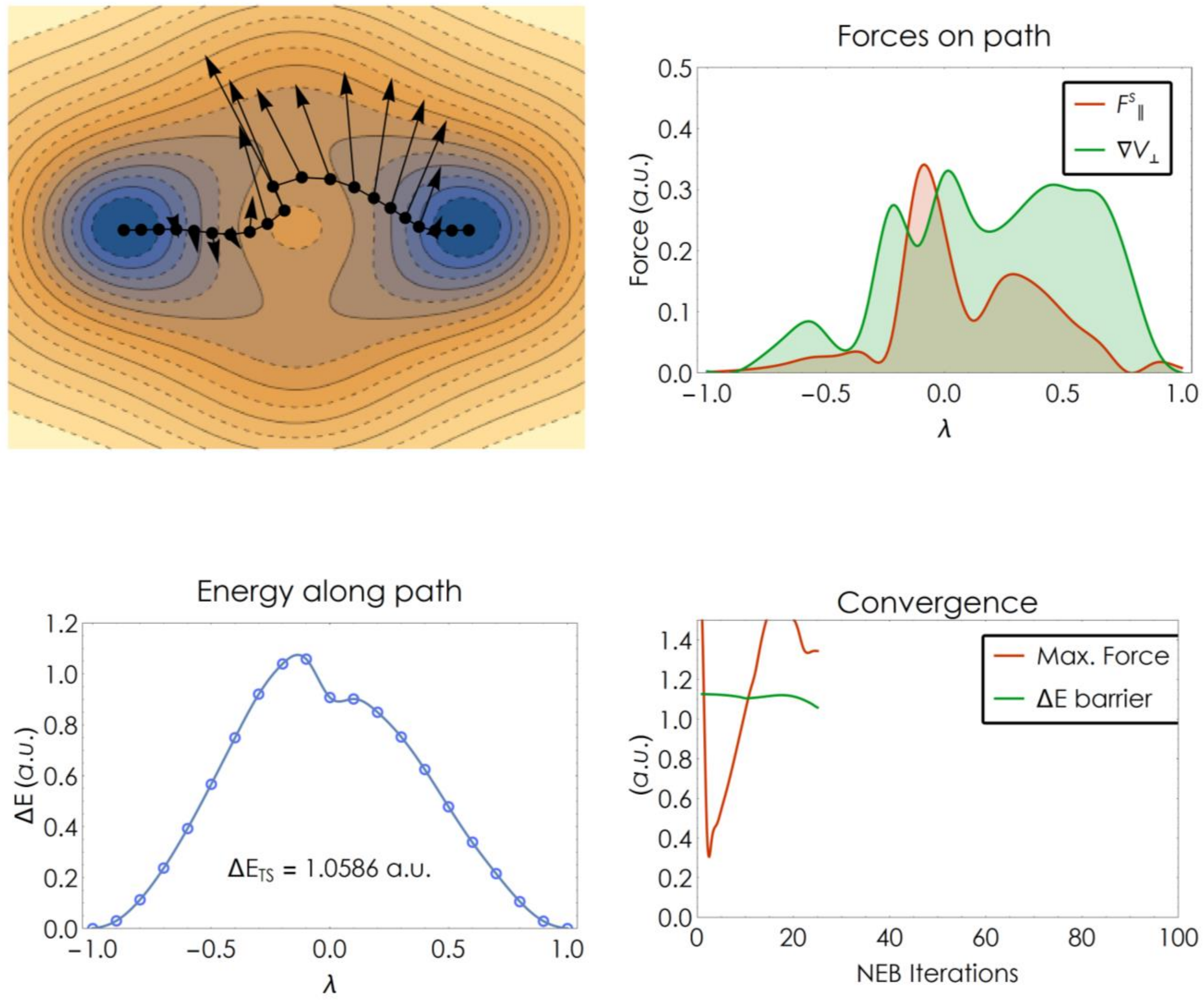

Supplementary Figure 6| Example of NEB convergence. On running the program Simple NEB.nb, panel **a** is the path (black dots connected with solid black lines) after 25 NEB iterations on an example 2-dimensional potential energy surface given by the Supplementary Equation (1) and shown as equipotential surface contours, with force vectors plotted as black arrows. Panel **b** is the magnitude of two components of the forces on the path (elastic band force is red, gradient force is green). Panel **c** is the current energy profile of the path and transition state energy, and panel **d** is the convergence of the forces and the energy barrier as a function of iterations, up to 25 iterations.

a

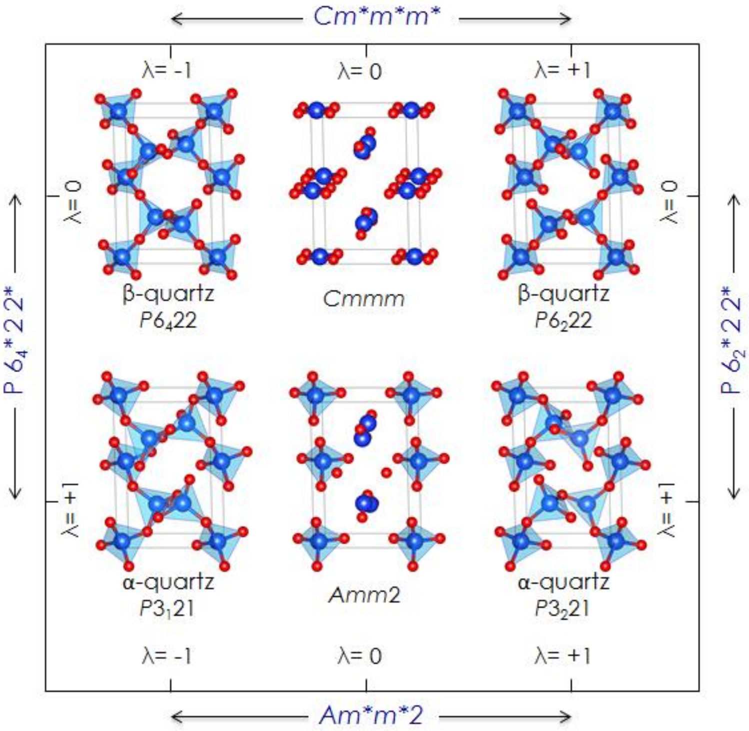

b

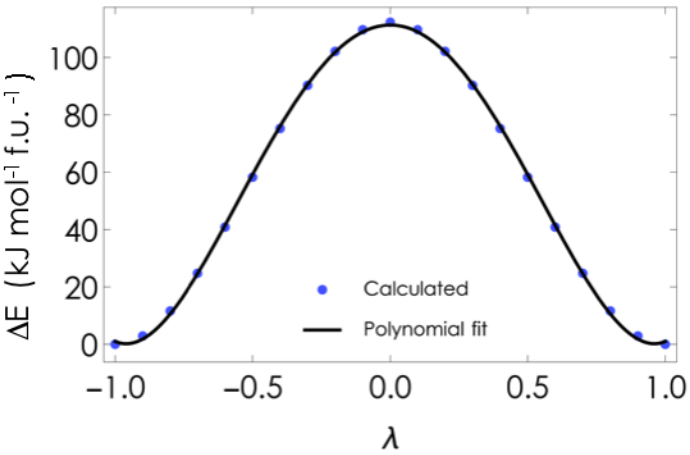

Supplementary Figure 7| Distortion symmetry in quartz pathways. In panel **a,** some distortion pathways and their distortion symmetry between left and right handed variants of α-quartz and β-quartz are given. The crystal structures at λ=-1, 0 and +1 are given for each, along with their symmetries. The distortion symmetries for four possible pathways between them are given along the edges of the rectangular box. Panel **b** plots the energy of the $P6_4$*22* example distortion as a function of λ; this path runs between two variants of left-handed α-quartz

through a transition state of β-quartz. The blue circles are the NEB calculations and the solid black line is the symmetrized fit.

Supplementary Note 1 - Connection between distortion symmetry and representation analysis

We will further establish the connection between distortion symmetry and representation analysis with the example of atomic displacements of a water molecule, H_2O, with conventional symmetry *mm*2 (C_{2v}) as depicted in Supplementary Figure 1a. Using the irreducible representations of mm2, we can construct a symmetry-adapted basis for the atomic displacements of H_2O. Our chosen basis is depicted in Supplementary Fig. 1a. Each of our basis modes correspond to a distortion path that is constructed by linearly scaling the displacements by λ, as depicted in Fig. 1h. The irrep carried by the span of each mode in our basis is labeled along with the symmetry of the corresponding distortion. The A_1 distortions have mm2 symmetry. The A_2, B_1, and B_2 distortions have *m***m**2, *m***m*2*, and *mm**2* symmetries, respectively. The properties of *mm*2 allow for this simple correspondence between one-dimensional irreps and distortion groups. In general though, such correspondences can be more complex when 2- or higher-dimensional irreps exist. For example, the ammonia molecule, NH_3, has 3*m* symmetry. 3*m* has a 2-dimensional irrep, denoted E. This irrep is carried by the linear span of V_1 and V_2 shown in Supplementary Figure 1b. If we construct distortions by linearly scaling V_1 and V_2 by λ, then the resulting distortions have symmetry *m* and *m** respectively. Thus, knowing the irreducible components of a set of displacements is not equivalent to knowing the symmetry of the corresponding distortion. The symmetry of distortions constructed from linear combinations of V_1 and V_2 is shown in Supplementary Fig. 1c. While most of this space (orange region) does have the kernel symmetry of 1, one can see specific linear combinations of V_1 and V_2 (black lines) which give rise to *m* and *m** symmetries. Complexities of representation analysis, such as in 2- or higher-dimensional irreps, are avoided by using distortion symmetry. For many problems, distortion symmetry offers a simple and elegant alternative to traditional representation analysis.

An improper ferroelectric antiferromagnet, $YMnO_3$, distorting from one ferroelectric domain, α^+ at λ=-1 to the opposite domain α^- at λ=+1 exhibits a distortion symmetry of $P6_3/m^*cm$ (Fig. 5c). This is also effectively the distortion implied by Fennie and Rabe[1] in studying the $P6_3/mmc$ parent structure. It is also an interesting case in terms of the relationship between distortion symmetry and representation analysis. Fennie and Rabe identify an unstable K_3 phonon mode of the $P6_3/mmc$ parent structure as driving the improper ferroelectric transition. K_3 is a 2D irrep and thus corresponds to two order parameters. Depending on order parameter direction, a perturbation that transforms as K_3 could result in $P6_3cm$, $P\bar{3}c1$, or $P3c1$ type symmetry. $P6_3cm$ and $P\bar{3}c1$ correspond to 1D subspaces of K_3 and their transformation properties should require that the corresponding distortions have $P6_3/m^*cm$ and $P6_3^*/m^*cm^*$ symmetry respectively. $P3c1$ is the kernel, the lowest possible symmetry achievable with a perturbation that transforms as K_3; it is the symmetry of a general point in the 2D order parameter space of K_3. Distortions corresponding to these points should have $P\bar{6}^*c2^*$ symmetry. In the $YMnO_3$ case, the order parameter direction corresponds to a distortion group of $P6_3/m^*cm$ and the coupled modes should not change this. We note the considerable complexity of describing the symmetry of this distortion with representation analysis (*i.e.* the last few sentences) versus comparative simplicity of the distortion symmetry classification (*i.e.* $P6_3/m^*cm$). This is an advantage of distortion symmetry over conventional representation analysis. We also note that giving the primary irrep of a distortion is *not* equivalent to giving a distortion group, just as is the case with magnetic symmetry[2], because K_3 actually corresponds to three different types of distortion symmetry: $P6_3/m^*cm$, $P6_3^*/m^*cm^*$, and $P\bar{6}^*c2^*$ depending on the direction of the order parameter.

Finally, we note one way in which the representations of distortion groups can be applied. The path depicted in Fig. 3a and in Supplementary Fig. 2 for an oxygen atom diffusion across a C_6 ring have m^*m2^* distortion symmetry. The character table for the m^*m2^*

group is given in Table 1. If we consider perturbations that displace the oxygen atom and not the carbon atoms, the 21 dimensional space of perturbations of the path (7 images across the path times 3 degrees of freedom for oxygen per image) carry a representation of *m**m*2* with the following irreducible components: 7 Γ_1 + 3 Γ_2 + 7 Γ_3 + 4 Γ_4. Using a symmetry-adapted basis, this can be decomposed into four symmetry invariant subspaces:

- the span of { Δx(-1)- Δx(1), Δz(-1)+ Δz(1), Δx(-2/3)- Δx(2/3), Δz(-2/3)+ Δz(2/3), Δx(-1/3)- Δx(1/3), Δz(-1/3)+ Δz(1/3), Δz(0)} which carries 7 Γ_1,
- the span of {Δy(-1)- Δy(1), Δy(-2/3)- Δy(2/3), Δy(-1/3)- Δy(1/3)} which carries 3 Γ_2,
- the span of {Δx(-1)+ Δx(1), Δz(-1)- Δz(1), Δx(-2/3)+ Δx(2/3), Δz(-2/3)- Δz(2/3), Δx(1/3)+ Δx(1/3), Δz(-1/3)- Δz(1/3), Δx(0)} which carries 7 Γ_3, and
- the span of {Δy(-1)+ Δy(1), Δy(-2/3)+ Δy(2/3), Δy(-1/3)+ Δy(1/3), Δy(0)} which carries 4 Γ_4.

where Δx(L) is the unit displacement of the oxygen atom in the image at λ=L along x (or y or z). These four subspaces correspond to symmetry breaking to *m**m*2*, 2*, *m*, and *m** respectively (these are the kernels of each irrep). Just as an ordinary symmetry-adapted basis for a static structure would put the force constants matrix in a block diagonal form, this basis will do so as well for the generalization force constants matrix that includes the nudged elastic band forces on the path; this would have blocks of 7, 3, 7, and 4 rows corresponding to each of the four subspace specified above. For this particular path, the first three blocks should be positive definite (stable, similar to having positive squared frequency with phonons of static structures). The final block, corresponding to the Γ_4 irrep, has one or more negative eigenvalues, indicating instability, that would then lead to a minimum energy pathway.

Supplementary Note 2: Additional analysis of NEB calculations for O diffusion across C_6 ring.

The initial path created by linearly interpolating between $\lambda = -1$ and $\lambda = +1$ has m^*m2^*, or more specifically $m_x^*m_y2_z^*$ symmetry where the subscripts represent axis associated with the operation: m_x^* is a starred mirror whose normal is along x, m_y is a mirror whose normal is along y, and 2_z^* is a starred two-fold axis along z (see compass on lower left in Supplementary Figure 2). Applying Neumann's principle to the force on the oxygen atom gives the following results:

- m_y $F_y(\lambda) = -F_y(\lambda) = F_y(\lambda)$, hence $F_y(\lambda) = 0$ (see **red** in Supplementary Fig. 2)
- m_x^* $F_x(\lambda) = -F_x(-\lambda) = F_x(\lambda)$, hence $F_x(\lambda)$ is an odd function of λ (see **blue**)
- 2_z^* $F_z(\lambda) = F_z(-\lambda) = F_z(\lambda)$, hence $F_z(\lambda)$ is an even function of λ (see **green**)

Clearly, the forces on both the initial and converged path are consistent with these symmetry predictions in Supplementary Figure 2. The forces of one iteration are used to update the positions for the next iteration, thus guaranteeing that the $m_x^*m_y2_z^*$ symmetry cannot be broken in any subsequent iteration.

In Supplementary Figure 3, we deliberately break the $m_x^*m_y2_z^*$ symmetry with a sinusoidal perturbation (the green curve is exaggerated; the maximum displacement was 0.1 Angstrom). Note that the forces on the initial path are similar to the unperturbed case, because the perturbation is small, but slightly break the previous symmetry. The perturbation is such that the path retains 2_z^* symmetry. Again applying Neumann's principle to the force on the oxygen atom gives the following results:

- 2_z^* $F_y(\lambda) = -F_y(-\lambda) = F_y(\lambda)$, hence $F_y(\lambda)$ is an odd function of λ (see **red**)
- 2_z^* $F_x(\lambda) = -F_x(-\lambda) = F_x(\lambda)$, hence $F_x(\lambda)$ is an odd function of λ (see **blue**)
- 2_z^* $F_z(\lambda) = F_z(-\lambda) = F_z(\lambda)$, hence $F_z(\lambda)$ is an even function of λ (see **green**)

Again, this is consistent with the forces on both the initial and converged path in Supplementary Fig. 3. The 2_z^* symmetry in the initial guess prevents NEB iterations from finding a significantly lower transition state (TS).

In Supplementary Figure 4, we deliberately break the m_x*$m_y$$2_z$* symmetry to trivial symmetry (the green curve is exaggerated; the maximum displacement was 0.18 Angstrom). Note that the forces on the initial path are similar to the unperturbed case, because the perturbation is small, but slightly break all previous symmetry. NEB iterations drive the path to the much lower energy *m** path seen in Figure 3d of the main text.

Supplementary Figure 5 shows two other ways of breaking *m***m*2* symmetry. We expect that NEB would drive the *m** initial path (Supplementary Fig. 5a) to the same low energy *m** path as seen in Figure 3d of the main text. The *m* initial path (Supplementary Fig. 5b) should not be able to converge to the low energy *m** path because *m* is not a subgroup of *m** and NEB iterations must conserve distortion symmetry.

Supplementary Note 3: Simple example to demonstrate the effect of distortion symmetry on NEB convergence

In the provided Mathematica Notebook file, Simple_NEB.nb, an example 2D potential energy surface (PES) is described and a simple implementation of the nudged elastic band (NEB) method is included. This PES is given as:

$$-4.07144 + 0.2e^{-x^2-4y^2} + \mathrm{Cos}[x]\mathrm{Cos}[y] + \mathrm{Cosh}[\tfrac{x}{2}] + 3\mathrm{Cosh}[\tfrac{y}{2}] \qquad (1)$$

"Simple_NEB.nb" contains dynamic and interactive plots that show what happens to the initial guess path as the NEB method iterates. Supplementary Figure 6 shows an example of what it might look like starting from a path with trivial symmetry after 25 iterations.

Using this implementation and PES, we tested the idea that applying distortion symmetry should result in a more rapid convergence of the NEB code. Our implementation was based on the explanation of the Nudged Elastic Band method given by Jónsson et al. [3]. Starting from a straight path, we generated 100,000 initial paths with trivial symmetry and 100,000 initial paths with *m** symmetry, which are the conventional symmetry and distortion symmetry, respectively, of the MEPs in this example. The details of how these were randomly generated are in the Simple_NEB.nb file. For each initial path, we ran our NEB implementation until the forces fell below a chosen convergence threshold. The results are summarized in Fig. 3f. Note that convergence is considerably more rapid with distortion symmetry in this example. The conventional symmetry paths typically took more than twice as long; the average number of iterations was about 443.8 for conventional symmetry and 190.8 for distortion symmetry. Symmetrizing using the correct distortion symmetry reduced the number of NEB iterations needed in 98.97% of our test cases and by a factor of 2.3 on average. Also note that if a straight path was provided as the initial guess, convergence to a MEP would not be possible in this example. Consequently, at least for this example, understanding distortion symmetry is crucial for achieving good results.

Supplementary Note 4: Examples of distortion symmetry in quartz

Supplementary Figure 7 shows the example of quartz, a common crystal that is found in left- and right- handed configurations, and is commonly used in watches and clocks as a crystal oscillator by using its piezoelectric effect. Trigonal α-quartz transforms into hexagonal β-quartz at 573°C, into hexagonal β-tridymite at 870°C and to cubic β-cristobalite at 1470°C. If β-quartz is considered a parent, the distortion to α-quartz has $P6_2$*22* symmetry for left-handed quartz and $P6_4$*22* symmetry for right-handed quartz (this is the distortion depicted in Fig. 5a of the main text). Note that $\lambda = +1$ and $\lambda = -1$ are different variants of α-quartz with β-quartz structurally intermediate between the two. In this instance, 1* does not reverse the handedness of the structures. However, if an appropriate parent is chosen, one can also transform between left- and right-handed α-quartz, as well as between left- and right- handed β-quartz. For α-quartz, our path has *Am***m**2 symmetry. For β-quartz, our path has *Cm***m***m** symmetry. This demonstrates a potentially surprising property of 1*, namely, that for carefully selected paths, 1* can reverse the handedness of a crystal. However, our $\lambda = 0$ structure is clearly not physically reasonably for either the *Am***m**2 symmetry path or the *Cm***m***m** symmetry path so it is very unlikely that it could be activated experimentally in practice. Other choices of paths are possible with the same or different distortion symmetries so there may be other paths with more reasonable transition states. Supplementary Figure 7b shows that, as with the examples given in the main text, the energy of the $P6_4$*22* distortion is symmetric with respect to λ due to the starred symmetry.

Supplementary Table 1: Examples of published studies that could have benefitted from the application of distortion groups. Second column lists figures with relevant starred symmetry implications within the publications referenced in the first column.

Reference	Figures	Description and notes
[4]	3,4,5,6	Stacking faults in olivine. All figures that should be symmetric due to starred symmetry show some degree of asymmetric error, Fig. 6 in particular. In part, this is due to having an even number of image.
[5]	3	Both Fig. 3a and 3b should be symmetric due to starred symmetry. Fig. 3a is a particularly extreme example of asymmetry error. The path taken in Fig. 3b is unstable and is balanced by symmetry as discussed in the main text.
[6]	3	Diffusion of Li and Mg in V_2O_5. The energy profiles show significant asymmetry error.
[7]	4a,c	Calculated energy for three different diffusion pathways for Li in V_2O_5 using NEB. Paths A and C have starred symmetry and thus figures 4a and 4c are symmetric. Path B does not have starred symmetry and thus figure 4b is asymmetric.
[8]	8b,c	Calculated energy of Li diffusion pathway in TiO_2 using NEB. The pathways for Fig 8b and 8c have starred symmetry and thus the energy plot is symmetric. Fig 9 depicts this pathway.
[9]	3	NEB study of diffusion of tungsten adatom on tungsten cluster surface. Figure 3 shows symmetric energy due to starred symmetry.
[10]	1c and 3a-d	Energy versus slip in magnesium alloys. Energy is symmetric for prismatic slip in Figure 1c due to starred symmetry. Symmetric energy in Figure 3a and 3b due to starred symmetry. The restoring force is antisymmetric in Figure 3c and 3d due to starred symmetry.
[11]	6	Diffusion path of MgO/O_3 cluster in MgO. Figure 6 is symmetric due to starred symmetry, but has small asymmetric errors.
[12]	2,3,4, and 5b	Constrained nudged elastic band calculation of the Peierls barrier with atomic relaxations. There are small but apparent deviations from symmetry (numerical errors).<br>Fig. 5b shows only half of the pathway, due to symmetry.
[13]	7	Stacking faults in Ni. Figure 7b (Theta=0.0) and 7c(Theta=0.0) are antisymmetric due to symmetry.
[14]	4a	N diffusion in fcc Fe. Fig. 4a is symmetric due to starred symmetry.
[15]	3	Carbon diffusion in supersaturated ferrite. Figure 3 ($x_c = 0$) is symmetric due to starred symmetry.
[16]	2,3	Ad-atom diffusion on copper surface. Figs. 2 and 3 are symmetric due to starred symmetry.
[17]	N/A	Cu diffusion in cristobalite. None of these figures are symmetric due to starred symmetry. Some are nearly symmetric barriers, but not exact. This is an example that shows that apparent symmetry in energy plots is not necessarily due to distortion symmetry (and therefore not exact).
[18]	2,3	An ab initio study of the effect of charge localization on oxygen defect formation and migration energies in magnesium oxide. Figs. 2 and 3 are symmetric due to starred symmetry.
[19]	3	Vacancy diffusion pathway, CI-NEB. Fig. 3 is symmetric due to starred symmetry.
[20]	4	H-atom relay reactions in real space. Fig. 4 is symmetric due to starred symmetry.
[21]	2	Stacking fault energy. Figure 2 is symmetric due to starred symmetry.
[22]	3,4,5	Stacking fault energy in Mg and Mg-Y alloys. Figs. 3, 4, and 5 are symmetric due to starred symmetry.
[23]	4	Diffusion in aluminum. Figure 4 is symmetric due to starred symmetry.

24	N/A	Lithium intercalation in TiO2-B. Shows the result of reversed pathways, e.g. Fig. 5.
25	2	Arrows depicting PF_5 pseudorotation in Fig. 2. As noted in the main text, these arrows are related by symmetry.
26	2a	Diffusion of oxygen in La_2CoO_4. Figure 2b shows Initial to Saddle images but not Saddle to Final, implying the intuitive application of symmetry.
27	7	Diffusion in Li_3N. Pathways depicted in Fig. 6. Figure 7 is symmetric due to starred symmetry.
28	3	Li diffusion in TiO_2. Figure 3 is symmetric due to starred symmetry.
29	5	Li diffusion on B_8C_{24} and $B_{24}C_{12}$. The path on B_8C_{24} has starred symmetry and thus the energy plot in Fig. 5c should be symmetric but is not, presumably due to errors. The path on $B_{24}C_{12}$ does not have starred symmetry and the asymmetry of Fig. 5d is consistent with this.
30	3e	O diffusion in La_2CoO_4. Figure 3e is symmetric due to starred symmetry.
31	5	NEB, molecular transitions. Figure 5 is symmetric due to starred symmetry.
32	N/A	Oxygen diffusion in lanthanum<br>silicate. Very interesting pathway from O5-0 to symmetry equivalent O5-0 site depicted in Fig. 9 and Fig. 10. Shows how equivalence of initial and final states (as noted in the captions) does not guarantee symmetry. This can be seen from the labels given along the O5-0 to O5-0 pathways, e.g. in Fig. 10 the pathway goes from O5-0 to O5-s2 to O5-s1 to O5-0. Because O5-s2 and O5-s1 are inequivalent, it is impossible to superimpose this pathway with its reverse and therefore there is no starred symmetry.
33	7	Migration pathway for Li in $LiFePO_4$. Fig.5 also depicts two different kinds of hops that occur in Li_xCoO_2, we note that the first (Fig. 5a) has starred symmetry but the other does not.
34	2	Diffusion of hydrogen atom on MoS_2. Figure 2 is symmetric due to starred symmetry.
35	N/A	Fig. 6 shows another example of a minimum energy pathway that is not superimposable with its reverse and therefore does not contain starred symmetry.
36	5a,b	Various migration pathways in Li_2MnO_3. Path 3 and 4 have starred symmetry, the rest do not.
37	3,4	Mg diffusion in $Mg_{0.5}FeSO_4F$. L1 and L2 pathways (Fig. 3) both have starred symmetry. Small deviations from this symmetry are apparent in Fig.4, *e.g.* the 5th and 13th points of Fig. 4a should be at the same height. Presumably this is due to errors in the calculations.
38	4	Na migration path on MoS2.<br>Very clear example with superimposed images showing the pathway. Both pathways in Figure 4 have starred symmetry.
39	7	Li diffusion in $LiVOPO_4$. Figure 7 is symmetric due to starred symmetry.
40	3	Na diffusion in $NaCoO_2$. Figure 3 is symmetric due to starred symmetry.
41	1c,d and 3	Figure 3 shows the expected diffusion path of Li in $LiFePO_4$. Depicted as continuous motion. Figure 1c,d show two different pathways. Both have clear starred symmetry. Considering only the finite structures depicted, 1c has m*m2* symmetry and 1d has 2*/m symmetry.
42	3a,d,e	Diffusion on Al_2O_3 surface. Fig. 3a,d, and e are symmetric due to starred symmetry.
43	2,4,6	Figs. 2, 4, and 6 are symmetric due to starred symmetry.

44	4b	Pathways in Fig. 4a and c have equivalent endpoints, but do not have starred symmetry (the approximate symmetry of the energy in Fig. 4c is not due to starred symmetry). The pathway in Fig. 4b should have starred symmetry but there are clear deviations in the energy (note 2nd and 2nd to last points). The starred symmetry suggests this is an error, maybe due to using an even number of images.
45	2,3,4	Adatom Diffusion on Fe surfaces. Small asymmetry in Fig 2 due to choosing an even number of points.
46	8	Diffusion in LaCoO3. Fig. 8c is interesting in the context of distortion symmetry because μA and μB are related by a starred operation and so the should be opposite with respect to the reaction coordinate and meet in the middle. This is consistent with the figure except they do not meet in the middle. This is either an error or an interesting case of magnetism breaking starred symmetry.
47	2	MedeA Transition State Search Datasheet. Shows an example of applying the MedeA Transition State Search module to the migration of a Pd_4 cluster on a MgO(001) surface. Figure 2 is symmetric due to starred symmetry.
48	2d,e (red and blue),f (red and blue)	Diffusion in $Li_4Ti_5O_{12}$, $Li_7Ti_5O_{12}$, and $Na_6LiTi_5O_{12}$. Fig. 2d,e (red and blue),f (red and blue) are symmetric due to starred symmetry.
49	3	NEB for studying switching of magnetic domains, uniform rotation versus domain wall motion. Data from Dittrich, R. et al. Energy Barriers in Magnetic Random Access Memory Elements. IEEE Trans. Magn. 39, 2839–2841 (2003). Fig. 3 is symmetric due to starred symmetry.
50	3 (red)	Stacking faults in Ni, Al, and Cu. Fig. 3 (red) is symmetric due to starred symmetry.
51	10	Pseudo-rotation of 1,2,3-$F_3C_6H_3^-$. In Figure 10b, starred symmetry has the consequence of making F_2 symmetric and F_1 and F_3 mirror images. This is similar to the PF_5 pseudorotation and bond lengths example given in the main text of our work.
52	5	Diffusion of Na and Li ions in $Na_{1.5}VPO_5F_{0.5}$ and $LiNa_{0.5}VPO_5F_{0.5}$. Fig. 5b and 5d give very clear depictions of the pathways. Fig. 5c and 5e are symmetric due to starred symmetry.
53	1	Fig. 1 is symmetric due to starred symmetry.
54	5	Fig. 5 is symmetric due to starred symmetry.
55	3	Diffusion in NiAl3. Fig. 3 is a comparison of NEB and constrained atom (CA) methods. Both show small asymmetry errors. Fig. 3 is symmetric due to starred symmetry.
56	1	Diffusion in Ti. Figure 1 is symmetric due to starred symmetry.
57	1,2	Li diffusion in olivine phosphates (FePO4 and LiFePO4). All paths with plotted energies are symmetric due to starred symmetry.
1	2,4	The distortions in this paper come from following the normal modes of a parent structure. Figure 4 is antisymmetric because of the starred symmetry.
58	3a	Figure 3a is symmetric due to starred symmetry.

Supplementary References

1. Fennie, C. J. & Rabe, K. M. Ferroelectric transition in YMnO3 from first principles. *Phys. Rev. B - Condens. Matter Mater. Phys.* **72,** 100103 (2005).

2. Perez-Mato, J. M., Ribeiro, J. L., Petrícek, V. & Aroyo, M. I. Magnetic superspace groups and symmetry constraints in incommensurate magnetic phases. *Journal of Physics: Condensed Matter* **24,** 163201 (2012).

3. Jonsson, H., Mills, G. & Jacobsen, K. W. in *Classical and Quantum Dynamics in Condensed Phase Simulations - Proceedings of the International School of Physics* 385–404 (1998). doi:10.1142/9789812839664_0016

4. Durinck, J., Legris, A. & Cordier, P. Pressure sensitivity of olivine slip systems: First-principle calculations of generalised stacking faults. *Phys. Chem. Miner.* **32,** 646–654 (2005).

5. Dai, Y., Ni, S., Li, Z. & Yang, J. Diffusion and desorption of oxygen atoms on graphene. *J. Phys. Condens. matter* **25,** 405301 (2013).

6. Zhou, B., Shi, H., Cao, R., Zhang, X. & Jiang, Z. Theoretical study on the initial stage of Magnesium battery based on V2O5 cathode. *Phys. Chem. Chem. Phys.* **16,** 18578–18585 (2014).

7. Ma, W. Y., Zhou, B., Wang, J. F., Zhang, X. D. & Jiang, Z. Y. Effect of oxygen vacancy on Li-ion diffusion in a V2O5 cathode: a first-principles study. *J. Phys. D. Appl. Phys.* **46,** 105306 (2013).

8. Morgan, B. J. & Watson, G. W. GGA+U description of lithium intercalation into anatase TiO2. *Phys. Rev. B - Condens. Matter Mater. Phys.* **82,** 144119 (2010).

9. Yang, J., Hu, W. & Tang, J. Surface self-diffusion behavior of individual tungsten adatoms on rhombohedral clusters. *J. Phys. Condens. Matter* **23,** 395004 (2011).

10. Tsuru, T. *et al.* Solution softening in magnesium alloys: the effect of solid solutions on the dislocation core structure and nonbasal slip. *J. Phys. Condens. Matter* **25,** 022202 (2013).

11. Mulroue, J., Uberuaga, B. P. & Duffy, D. M. Charge localization on the hexa-interstitial cluster in MgO. *J Phys Condens Matter* **25,** 65502 (2013).

12. Gröger, R. & Vitek, V. Constrained nudged elastic band calculation of the Peierls barrier with atomic relaxations. *Model. Simul. Mater. Sci. Eng.* **20,** 035019 (2012).

13. Schusteritsch, G. & Kaxiras, E. Sulfur-induced embrittlement of nickel: a first-principles study. *Model. Simul. Mater. Sci. Eng.* **20,** 065007 (2012).

14. Wu, M. H., Liu, X. H., Gu, J. F. & Jin, Z. H. DFT study of nitrogen – vacancy complexions in (fcc) Fe. *Model. Simul. Mater. Sci. Eng.* **22,** 055004 (2014).

15. Lawrence, B., Sinclair, C. W. & Perez, M. Carbon diffusion in supersaturated ferrite: a comparison of mean-field and atomistic predictions. *Model. Simul. Mater. Sci. Eng.* **22,** 065003 (2014).

16. Samanta, A. & Weinan, E. Optimization-based string method for finding minimum energy path. *Commun. Comput. Phys.* **14,** 265–275 (2013).

17. Zelený, M. *et al.* Ab initio study of Cu diffusion in α-cristobalite. *New J. Phys.* **14,** 113029 (2012).

18. Mulroue, J. & Duffy, D. An ab initio study of the effect of charge localization on oxygen defect formation and migration energies in magnesium oxide. *Proc. R. Soc. London. A. Math. Phys. Sci.* **467,** 2054–2065 (2011).

19. Spiewak, P. & Kurzydlowski, K. J. Formation and migration energies of the vacancy in Si calculated using the HSE06 range-separated hybrid functional. *Phys. Rev. B - Condens. Matter Mater. Phys.* **88,** 195204 (2013).

20. Kumagai, T. *et al.* H-atom relay reactions in real space. *Nat. Mater.* **11,** 167–172 (2011).

21. Pizzagalli, L. *et al.* A new parametrization of the Stillinger-Weber potential for an improved description of defects and plasticity of silicon. *J. Phys. Condens. Matter* **25,** 055801 (2013).

22. Pei, Z. *et al.* Ab initio and atomistic study of generalized stacking fault energies in Mg and Mg-Y alloys. *New J. Phys.* **15,** 043020 (2013).

23. Ho, G., Ong, M. T., Caspersen, K. J. & Carter, E. a. Energetics and kinetics of vacancy diffusion and aggregation in shocked aluminium via orbital-free density functional theory. *Phys. Chem. Chem. Phys.* **9,** 4951–4966 (2007).

24. Panduwinata, D. & Gale, J. D. A first principles investigation of lithium intercalation in TiO2-B. *J. Mater. Chem.* **19,** 3931 (2009).

25. Berry, S. Correlation of rates of intramolecular tunneling processes, with application to some group V compounds. *J. Chem. Phys.* **32,** 933–938 (1960).

26. Kushima, A. *et al.* Interstitialcy diffusion of oxygen in tetragonal La2CoO(4+δ). *Phys. Chem. Chem. Phys.* **13,** 2242–2249 (2011).

27. Li, W. *et al.* Li+ ion conductivity and diffusion mechanism in α-Li3N and β-Li3N. *Energy Environ. Sci.* **3,** 1524 (2010).

28. Yildirim, H., Greeley, J. P. & Sankaranarayanan, S. K. R. S. The effect of concentration on Li diffusivity and conductivity in rutile TiO2. *Phys. Chem. Chem. Phys.* **14,** 4565 (2012).

29. Ye, X.-J., Liu, C.-S., Jia, R., Zeng, Z. & Zhong, W. How does the boron concentration affect hydrogen storage in lithium decorated zero- and two-dimensional boron-carbon compounds? *Phys. Chem. Chem. Phys.* **15,** 2507–13 (2013).

30. Han, J. W. & Yildiz, B. Mechanism for enhanced oxygen reduction kinetics at the (La,Sr)CoO3/(La,Sr)2CoO4+ hetero-interface. *Energy Environ. Sci.* **5,** 8598 (2012).

31. Kuhlman, T. S., Glover, W. J., Mori, T., Møller, K. B. & Martínez, T. J. Between ethylene and polyenes - the non-adiabatic dynamics of cis-dienes. *Faraday Discuss.* **157,** 193 (2012).

32. Matsunaga, K. & Toyoura, K. First-principles analysis of oxide-ion conduction mechanism in lanthanum silicate. *J. Mater. Chem.* **22,** 7265 (2012).

33. Islam, M. S. & Fisher, C. A. J. Lithium and sodium battery cathode materials: computational insights into voltage, diffusion and nanostructural properties. *Chem. Soc. Rev.* **43,** 185–204 (2014).

34. Cai, Y. *et al.* Constructing metallic nanoroads on a MoS2 monolayer via hydrogenation. *Nanoscale* **6,** 1691–1697 (2014).

35. Murugesan, S. *et al.* Wide electrochemical window ionic salt for use in electropositive metal electrodeposition and solid state Li-ion batteries. *J. Mater. Chem. A* **2,** 2194 (2014).

36. Gao, Y. *et al.* Improved electron/Li-ion transport and oxygen stability of Mo-doped Li2MnO3. *J. Mater. Chem. A* **2,** 4811 (2014).

37. Wu, J. *et al.* Tavorite-FeSO4F as a potential cathode material for Mg ion batteries: a first principles calculation. *Phys. Chem. Chem. Phys.* **16,** 22974–22978 (2014).

38. Su, J., Pei, Y. & Wang, X. Ab initio study of graphene-like monolayer molybdenum disulfide as a promising anode material for rechargeable sodium ion batteries. *RSC Adv.* **4,** 43183–43188 (2014).

39. Ling, C., Zhang, R. & Mizuno, F. Phase stability and its impact on the electrochemical performance of VOPO 4 and LiVOPO 4. *J. Mater. Chem. A* **2,** 12330 (2014).

40. Mo, Y., Ong, S. P. & Ceder, G. Insights into Diffusion Mechanisms in P2 Layered Oxide Materials by First-Principles Calculations. *Chem. Mater.* **26,** 5208–5214 (2014).

41. Nishimura, S. *et al.* Experimental visualization of lithium diffusion in LixFePO4. *Nat. Mater.* **7,** 707–711 (2008).

42. Milas, I., Hinnemann, B. & Carter, E. a. Diffusion of Al, O, Pt, Hf, and Y atoms on α-Al2O3(0001): implications for the role of alloying elements in thermal barrier coatings. *J. Mater. Chem.* **21,** 1447 (2011).

43. Marinica, M.-C. *et al.* Interatomic potentials for modelling radiation defects and dislocations in tungsten. *J. Phys. Condens. Matter* **25,** 395502 (2013).

44. Chang, H. *et al.* Single adatom dynamics at monatomic steps of free-standing few-layer reduced graphene. *Sci. Rep.* **4,** 6037 (2014).

45. Wang, C. *et al.* Single Adatom Adsorption and Diffusion on Fe Surfaces. *Journal of Modern Physics* **02,** 1067–1072 (2011).

46. Ritzmann, A. M., Pavone, M., Muñoz-García, A. B., Keith, J. A. & Carter, E. A. Ab initio DFT+U analysis of oxygen transport in LaCoO3: the effect of Co3+ magnetic states. *J. Mater. Chem. A* **2,** 8060 (2014).

47. MedeA Transition State Search Datasheet, http://www.materialsdesign.com/medea/transition-state-search

48. Sun, Y. *et al.* Direct atomic-scale confirmation of three-phase storage mechanism in Li4Ti5O12 anodes for room-temperature sodium-ion batteries. *Nat. Commun.* **4,** 1870 (2013).

49. Khvalkovskiy, A. V *et al.* Basic principles of STT-MRAM cell operation in memory arrays. *J. Phys. D. Appl. Phys.* **46,** 074001 (2013).

50. Van Swygenhoven, H., Derlet, P. M. & Frøseth, A. G. Stacking fault energies and slip in nanocrystalline metals. *Nat. Mat.* **3,** 399–403 (2004).

51. Schegoleva, L. N. & Beregovaya, I. V. Manifestation of Complicated Structure of Potential Energy Surfaces in Spectral and Chemical Properties of Haloarene Radical Ions. *Fluor. Notes* **2**(87) (2013).

52. Park, Y.-U. *et al.* Tailoring a fluorophosphate as a novel 4 V cathode for lithium-ion batteries. *Sci. Rep.* **2,** 704 (2012).

53. Wang, J., Ewing, R. C. & Becker, U. Average structure and local configuration of excess oxygen in UO2+x. *Sci. Rep.* **4,** 4216 (2014).

54. Tang, Z. K., Zhang, Y. N., Zhang, D. Y., Lau, W. M. & Liu, L. M. The stability and electronic properties of novel three-dimensional graphene-MoS2 hybrid structure. *Sci. Rep.* **4**, 7007 (2014).

55. Tingaud, D., Nardou, F. & Besson, R. Diffusion in complex ordered alloys: Atomic-scale investigation of NiAl3. *Phys. Rev. B - Condens. Matter Mater. Phys.* **81,** 174108 (2010).

56. Shang, S., Hector Jr., L. G., Wang, Y. & Liu, Z. K. Anomalous energy pathway of vacancy migration and self-diffusion in hcp Ti. *Phys. Rev. B* **83,** 224104(2011).

57. Dathar, G. K. P., Sheppard, D., Stevenson, K. J. & Henkelman, G. Calculations of Li-ion diffusion in olivine phosphates. *Chem. Mater.* **23,** 4032–4037 (2011).

58. Meng, X. *et al.* Direct visualization of concerted proton tunnelling in a water nanocluster. *Nat. Phys.* **11,** 235–239 (2015).
